# Overcoming Charge-Carrier Localization in Metal Chalcohalides

**DOI:** 10.1021/jacs.6c05142

**Published:** 2026-06-22

**Authors:** Bembe C. Mackintosh, Marcello Righetto, G. Krishnamurthy Grandhi, Thomas B. Haward, Noolu Srinivasa Manikanta Viswanath, Joshua R. S. Lilly, Siyu Yan, Jae Eun Lee, Snigdha Lal, Alan R. Bowman, Michael B. Johnston, Paola Vivo, Laura M. Herz

**Affiliations:** 1 Department of Physics, University of Oxford, Clarendon Laboratory, Parks Road, Oxford OX1 3PU, United Kingdom; 2 Department of Chemical Science, Università degli Studi di Padova, Via Marzolo 1, Padova I-35131, Italy; 3 Hybrid Solar Cells, Faculty of Engineering and Natural Sciences, 7840 Tampere University, P.O. Box 541, Tampere FI-33014, Finland; 4 Division of Materials Science and Engineering, 26716 Hanyang University, 222 Wangsimni-ro, Seongdong-gu, Seoul 04763, Republic of Korea

## Abstract

Effective charge-carrier transport is a key requirement of next-generation thin-film materials developed for solar cells. Perovskite-inspired materials (PIMs), including metal chalcohalides, show great promise as lead-free solar absorbers. However, intrinsic charge-carrier localization processes have frequently been reported to severely limit their transport properties. Recent research has thus focused on developing a rational understanding of this localization process and identifying strategies to eliminate it. Mixed-metal chalcohalides (A_2_BCh_2_X_3_) may offer promising solutions, combining enhanced chemical stability with promising optoelectronic properties. Here, we demonstrate how charge-carrier localization can be overcome through judicious chemical substitution in this family of materials. Upon changing the M­(II) cation on the A-site, the lattice symmetry shifts from the lower-symmetry monoclinic *P2*_1_ */c* phase in Pb_2_SbS_2_I_3_ to the higher-symmetry orthorhombic *Cmcm* phase in Sn_2_SbS_2_I_3_. Crucially, a rapid localization of charge carriers within the first few picoseconds of their generation is observed only for Pb_2_SbS_2_I_3_, whereas Sn_2_SbS_2_I_3_ maintains a longer-lived nanosecond photoconductivity. We attribute this observation to the higher electronic dimensionality of the *Cmcm* Sn_2_SbS_2_I_3_ structure, whose more symmetric lattice suppresses the charge-carrier localization dominating in the lower-dimensional *P2*_*1*_ */c* Pb-analogue. These findings establish a direct link between structural and optoelectronic properties in metal chalcohalides, demonstrating how facile chemical tuning can be harnessed to overcome charge-carrier localization in PIM absorbers for solar energy harvesting.

## Graphical abstract

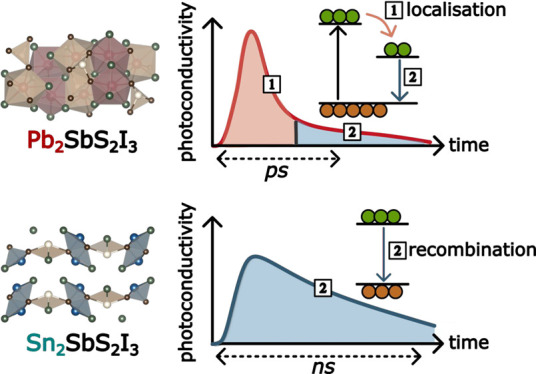

## Introduction

Perovskite-inspired materials (PIMs) are an emerging group of semiconductors aiming to overcome the stability and toxicity issues of lead-halide perovskites.^1–4^ Within the broad PIM family, chalcohalides and oxyhalides are among the most promising compositions.^5–7^ A wide variety of chalcohalides (often abbreviated as MChX or MOX, with M as the metal cation(s), and Ch/O and X as the chalcogen/oxygen and halide ions) have emerged as a promising class of PIMs, designed to inherit the benign defect chemistry and excellent optoelectronic characteristics of metal-halide perovskite (MHP).^6^ Partially oxidized cations of post-transition metals (M = Bi^3+^, Sb^3+^, Sn^2+^, Pb^2+^) provide the sought-after high-Z ns^2^ electronic configuration, while the combination of the mono- and divalent anions (Ch = O^2–^, S^2–^, Se^2–^ ; X = I^–^, Br^–^, Cl^–^) enables systematic tuning of both the band structure and the lattice framework.^7–9^ For example, bismuth oxyiodide (BiOI) has shown excellent performance in photo­(electro)­chemical cells, radiation detectors and indoor photovoltaics.^10–12^ Such promising defect-tolerant properties in BiOI originate from the hybridization of the stereochemically active 6s^2^ lone pair on Bi­(III) with the iodine p-orbital electrons, and the consequent antibonding character of the band edges.^6,13^ Furthermore, favorable charge-carrier transport properties, such as a fair mobility^14^ and nanosecond charge-carrier^15^ lifetime, have been reported for BiOI. However, its wide band gap (>2 eV) limits its applicability as a solar light harvester in photovoltaic devices.

Alternative heavy-pnictogen chalcohalide compositions, including SbSI, BiSI, and SbSeI, have been recently proposed and investigated for solar cell applications.^16,17^ In these materials, the contributions of higher-lying S 3p and Se 4p orbitals, compared with O 2p orbitals, yield more dispersive bands and narrower band gaps.^5^ Despite their promising band gaps, morphology issues have been reported for chalcohalide thin films, mainly caused by their needle-like 1D and platelet 2D growth.^16^ In solar cells, these uneven morphologies lead to low fill factors and open-circuit voltages, posing a major hurdle to the overall photovoltaic performance of heavy-pnictogen chalcohalides.^5,18,19^ Extending the compositional space to mixed-metal chalcohalides, thus including two different metallic cations, has also been proposed to enhance band gap tunability.^19^ The potential of this approach has been demonstrated for (Ag,Bi)­(S,I) thin films by the Simonov group, which reported a PIM record solar cell power conversion efficiency (PCE) of ∼5.5% for Ag_3_BiI_5.96_S_0.04_.^20^ In addition, the Kanatzidis group reported the first series of A_2_BCh_2_X_3_ chalcohalides in 2016, which feature both A­(II) (Sn^2+^, Pb^2+^) and B­(III) (Sb^3+^, Bi^3+^) cations.^21^ These materials have demonstrated highly promising incipient solar cell performances: Sn_2_SbS_2_I_3_ has a reported initial PCE of 4.04%,^22^ and excellent environmental stability,^22,23^ whereas the wider-band gap Pb analogue, Pb_2_SbS_2_I_3_ has achieved a slightly lower PCE of 3.12%.^24^ We note that the well-known instability of Sn^2+^ in conventional tin-halide perovskites does not straightforwardly apply to A_2_BCh_2_X_3_ compounds, for which the heteroleptic lattice environment with mixed sulfide-iodide coordination of the metal site fundamentally differs.^25^ Such differences are also evident from the excellent environmental stability of Sn_2_SbS_2_I_3_ which is consistent with the suppression of this oxidation pathway in the chalcohalide.^23^

Such A_2_BCh_2_X_3_ mixed-metal chalcohalides could crystallize in both orthorhombic (*Cmcm*/*Cmc2*_1_) and monoclinic (*P2*_1_ */c*) symmetry of infinite quasi-1D chain motifs, weakly coupled through halide polyhedra.^21,26^ Electronic-structure calculations reveal a direct band gap at the Γ point in the Brillouin zone, characterized by a flat valence band maximum (VBM) and a dispersive conduction band minimum (CBM) that accord small electron effective masses.^21,26^ Pb_2_BiS_2_I_3_ has a VBM dominated by the interaction between the anion p-orbitals, while the CBM is dominated by the Bi (p), I (p) and S (p) orbitals, notably missing the desired interaction with the cation-ns^2^ lone-pair of electrons.^13^ Sn_2_BiS_2_I_3_, on the other hand, includes such a contribution, with the VBM and CBM formed from a hybridized Sn (s), S (p), I (p) and Bi (p), giving the VB an antibonding character known to promote benign defect chemistry.^21^ Counter to the expected heavy-atom trend, replacing Pb with Sn has been reported to increase the band gap in related A_2_BCh_2_X_3_ bismuth analogues, attributed to the relativistic contraction of the Pb 6s^2^ orbitals, pushing the Sn 5s^2^ to a higher relative energy.^6,21^ In addition, Sn_2_SbS_2_I_3_ has been proposed to exhibit spontaneous polarization: *ab initio* calculations suggest that the reported centrosymmetric *Cmcm* phase represents an average over multiple polar *Cmc2*_1_ domains, driven by Sb-off centering, leading to a large spontaneous polarization (∼37 μC cm^–2^).^25^ It has been proposed that such effects yield efficient electron–hole separation.^27^

Interestingly, despite their promising performance in thin-film solar cells, these chalcohalides have, so far, been incorporated only in conjunction with mesoporous TiO_2_ scaffolds.^28^ While such mp-TiO_2_ can assist film formation and thus alleviate the microstructural issues prevailing in these materials, the lack of reports on chalcohalide-based planar heterojunctions could also indicate poor charge-carrier transport. Transport issues could potentially arise from ultrafast localization which has been reported for 3D and 2D double perovskite and Rudorffite metal halides as well as some ABZ_2_ compounds.^29–37^ Such charge-carrier localization manifests as a significant reduction in conductivity within picoseconds after pulsed-light absorption. The resulting localized states exhibit lower mobility and a less efficient, temperature-activated transport mechanism typically associated with small polarons, which may severely limit charge extraction in solar cells.^29,38–42^ However, to date, the extent to which such charge localization occurs in mixed-metal chalcohalides, and its link with composition and structure are still largely unknown.

In this work, we provide effective design criteria for eliminating ultrafast charge-carrier localization in chalcohalides. We contrast two mixed-metal chalcohalides, Pb_2_SbS_2_I_3_ and Sn_2_SbS_2_I_3_, combining structural and ultrafast optical probes to uncover the interplay between structure and charge-carrier transport. Careful analysis of X-ray diffraction patterns through Rietveld refinement reveals that Pb_2_SbS_2_I_3_ crystallizes in a monoclinic structure (*P2*_1_ */c*) with lower symmetry than the orthorhombic (*Cmcm*) Sn_2_SbS_2_I_3_. Interestingly, ultrafast measurements reveal that Pb_2_SbS_2_I_3_ undergoes rapid charge-carrier localization while Sn_2_SbS_2_I_3_ sustains a much longer-lived photoconductivity. These results demonstrate how substitution on the A-cation site in mixed-metal chalcohalides can modulate the charge-transport efficiency through symmetry transition. We propose that symmetry-driven changes in electronic dimensionality cause the observed differences in ultrafast charge-carrier dynamics, as reduced dimensionality has been suggested to lower the potential barriers to self-localization of charge carriers.^40^ Our findings will provide a useful guide to direct future research on chalcohalides and pave the way for efficient chalcohalide-based solar cells.

## Results and Discussion

Herein, we investigate thin films of A_2_SbS_2_I_3_ (A = Pb/Sn), deposited on a mesoporous TiO_2_ scaffold (to support uniform film formation; further discussion provided in the Supporting Information) supported by z-cut quartz substrates, using a double-annealing process, as previously reported^43^ and summarized in the Supporting Information. To gain insight into the structure and phase purity, we carried out X-ray diffraction (XRD). Structural models were refined through Rietveld refinements of XRD data, which are visualized in Figure 1a,b (for A = Pb and Sn respectively), with the corresponding experimental patterns and refinement fits shown in Figure 1c. For Pb_2_SbS_2_I_3_, the high intensity peaks at 2θ = 25° and 29.5° closely match the relative intensities for reported patterns of the monoclinic *P2*_1_ */c* space group,^44^ thus this phase was chosen as the basis for the refinement. For Sn_2_SbS_2_I_3_ (*Cmcm*), a secondary phase of Sb_2_S_3_ was identified, and the Rietveld refinement was carried out using both phases as a basis, consistent with previous reports of its coexistence with the primary phase (a more detailed discussion of the Rietveld refinement parameters can be found in the Supporting Information, Tables S1–S4).^22,43^ Rietveld refinement confirms that Pb_2_SbS_2_I_3_ adopts the lower-symmetry *P2*_1_ */c* structure, whereas Sn_2_SbS_2_I_3_ crystallizes in the higher-symmetry orthorhombic *Cmcm* phase. Further discussion of local structural parameters and distortion metrics is provided in Supporting Information Note 1.

**Figure 1:**
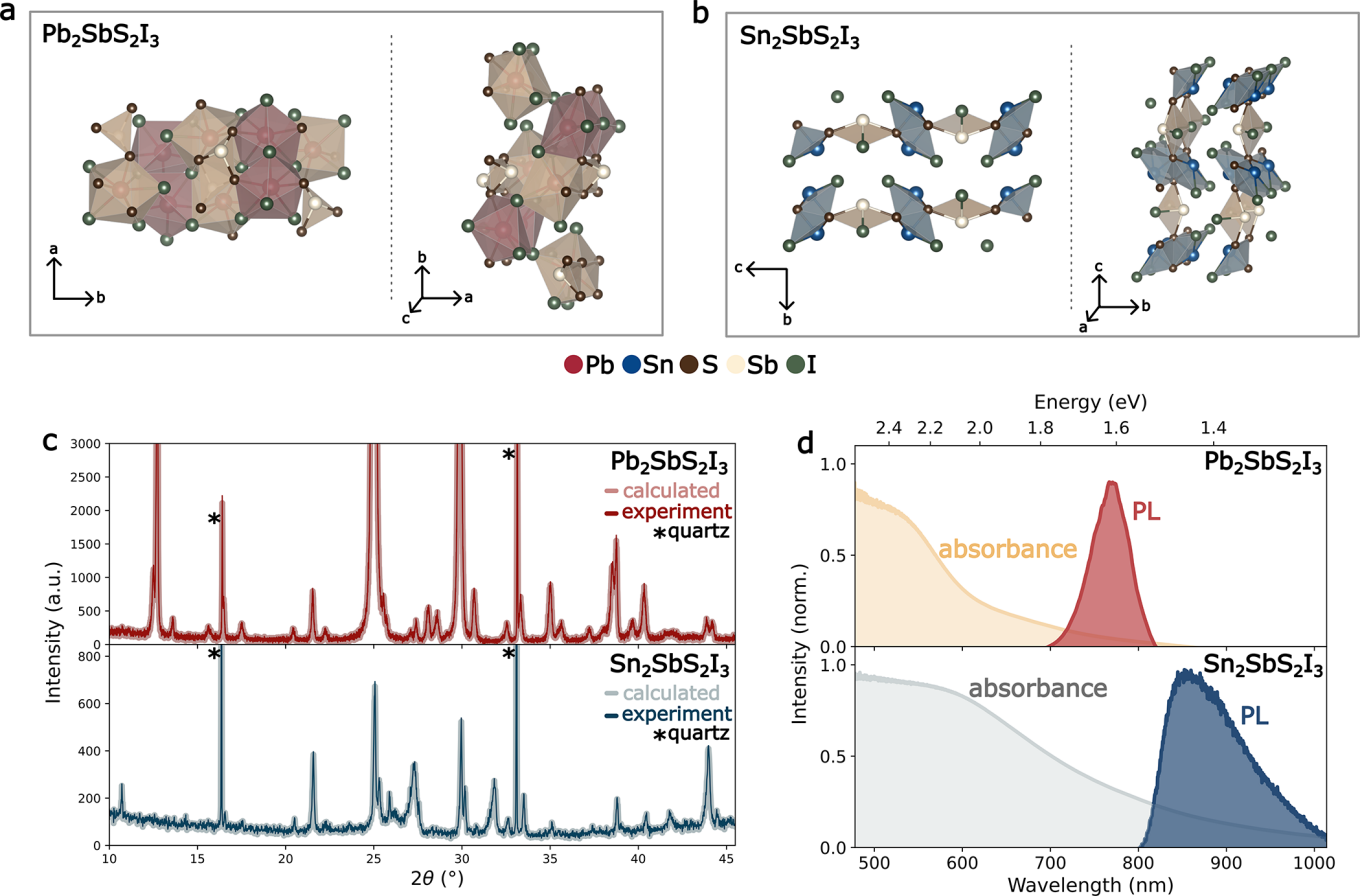
**| XRD, optical absorption and PL characterization of Pb- and Sn-based mixed-metal chalcohalides.** (a-b) Visualization of the unit cell of the crystal structure for Pb_2_SbS_2_I_3_ and Sn_2_SbS_2_I_3_, in the *P2*_1_ */c* and *Cmcm* space groups, respectively. (c) XRD patterns (full XRD patterns are provided in the Supporting Information, Figures S3 and S4) of thin films on mesoporous TiO_2_-coated quartz, together with the corresponding Rietveld refinement. Dark thin red and blue lines indicate experimental data, while thick shaded pink and gray lines represent the fitted refinement for Pb_2_SbS_2_I_3_ and Sn_2_SbS_2_I_3_, respectively. Asterisks denote peaks associated with the z-cut quartz substrate. XRD was performed using the Cu Kα_1_ line. (d) Optical absorption and PL emission spectra at room temperature (294 K). The PL spectrum for Pb_2_SbS_2_I_3_ was collected with a continuous-wave 3.1 eV laser excitation. Sn_2_SbS_2_I_3_ spectra were obtained with a pulsed 1.6 eV laser and a 1.5 eV long-pass filter in place to filter out scattered laser light, which also attenuates part of the emitted signal.

The linear absorption and photoluminescence (PL) spectra shown in Figure 1d reveal distinct optical properties despite their chemical similarity. Sn_2_SbS_2_I_3_ lacks a sharp absorption onset, instead the absorption increases gradually over a wide energy range, giving the appearance of a broad onset, consistent with previously reported spectra.^22,23,43^ This behavior primarily reflects a weak optical transition near the band edge: Kavanagh et al.^25^ reported a low transition dipole moment for the VBM → CBM transition (at 1.08 eV), which is Laporte-forbidden owing to the parity symmetry. As such, the observed rise in absorption around 1.4 eV reflects the energetic region where a number of interband transitions successively become allowed. This is consistent with the experimentally observed optical band gap of 1.4–1.5 eV for Sn_2_SbS_2_I_3_.^22,45^ Additional contributions from the coexistence of the Sb_2_S_3_ phase appear at slightly higher energies (1.6–1.8 eV),^46^ which, in combination with the anticipated static/dynamic disorder,^27^ makes the overall absorption onset appear even more gradual. Comparatively, Pb_2_SbS_2_I_3_ shows a much sharper rise in absorption around 1.8 eV, also in agreement with reported absorption spectra,^24^ representative of its relative phase purity and lower local distortion compared to that of its Sn analogue.

Both mixed-metal chalcohalides exhibit photoluminescence somewhat below their main absorption edge. For Pb_2_SbS_2_I_3_ a relatively strong PL intensity, centered around 1.6 eV following 3.1 eV CW excitation, is observed (Figure 1d), exhibiting a significant Stokes shift (∼0.2 eV).^21,24^ As previously reported for other PIMs,^29,32^ large Stokes shifts may indicate that the emission originates from a self-localized state. In contrast, Sn_2_SbS_2_I_3_ exhibits a lower Stokes shift but extremely weak PL which required excitation with a high-fluence (∼17 μJ cm^–2^, see the Supporting Information for details) pulsed laser at 1.6 eV to yield a broad peak centered at 1.5 eV. Such weak and broadened emission may reflect dominant nonradiative recombination in this more dynamically disordered lattice. The stronger stereochemically active 5s^2^ lone-pair of Sn^2+^ is expected to enhance electron–phonon coupling and excited-state lattice relaxation, which can broaden the emission while reducing radiative recombination probability.^13,47^ In addition, the weak, Laporte-forbidden band-edge transition further disfavors effective radiative decay.^25^

To investigate how the different crystal structures of these chalcohalides affect charge-carrier dynamics and transport, we employ ultrafast optical pump-terahertz probe (OPTP) and transient absorption (TA) spectroscopy with 3.1 eV pulsed excitation. While OPTP reflects the photoconductivity as a function of time after excitation, TA is mostly representative of the density of charge carriers. Figure 2a reveals that both Sn_2_SbS_2_I_3_ and Pb_2_SbS_2_I_3_ exhibit an instantaneous rise in photoconductivity (limited by the instrument response function), as typical for the generation of free charge carriers. Crucially, however, the subsequent photoconductivity decay is markedly different for the two materials. For Pb_2_SbS_2_I_3_ films, the THz photoconductivity signal drops by >80% of its original intensity on a picosecond time scale. The remnant photoconductivity decays on a slower time scale of ∼25 ps. Such observed THz photoconductivity decays, with a fluence-independent initial ultrafast decay followed by a slower decay in conductivity, have been widely reported for other PIMs and are the hallmark of ultrafast charge-carrier localization.^29,32,39,40^ Here, initially photogenerated free charge-carriers are rapidly transferred, within a few picoseconds, to a localized state with lower charge-carrier mobility and temperature-activated transport, reflective of small polarons.^39^ Notably, the reduced charge-carrier mobility of these localized states underlies the observed dramatic decay in the OPTP response. Conversely, such ultrafast charge-carrier localization is absent in the THz photoconductivity transients recorded for Sn_2_SbS_2_I_3_ thin films, which instead display a slower decay occurring over hundreds of picoseconds.

**Figure 2:**
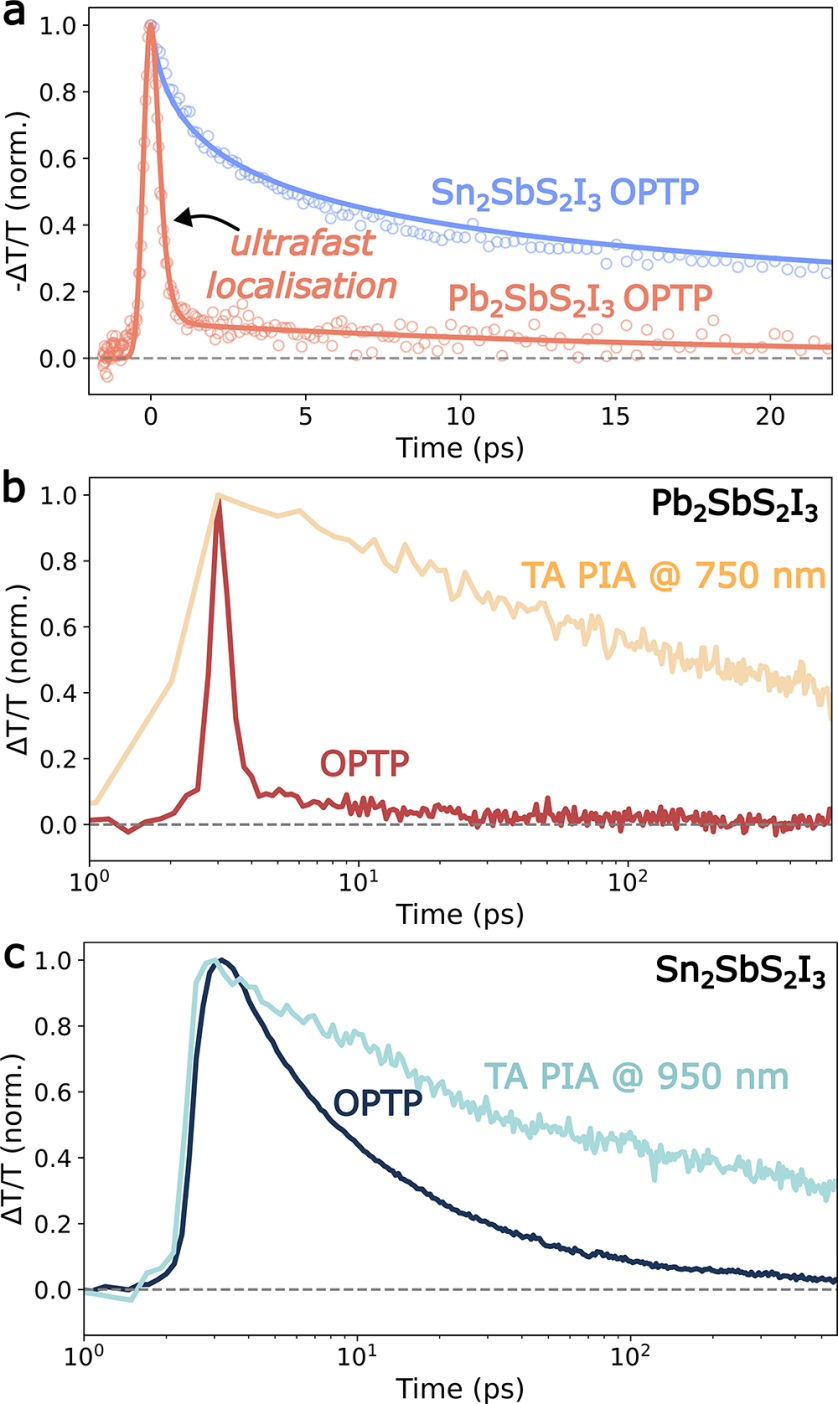
**| Comparison of ultrafast transient photoconductivity and absorption dynamics for Sn**_2_ **SbS**_2_ **I**_3_ **and Pb**_2_ **SbS**_2_ **I**_3_. (a) OPTP data recorded at 68 and 56 μJ/cm^2^ for Sn_2_SbS_2_I_3_ and Pb_2_SbS_2_I_3_ films, respectively. Experiment carried out with a pulsed excitation at 3.1 eV. (b-c) Comparison between the *in situ* photoconductivity (OPTP) and charge-carrier dynamics (PIA) for Pb_2_SbS_2_I_3_ (red/orange) and Sn_2_SbS_2_I_3_ (dark blue/light blue), respectively. TA data recorded at the wavelength corresponding to the absorption onset in each material as indicated.

To ensure that we can correctly disentangle effects of a mobility reduction from those arising from charge-carrier recombination, we further recorded TA data. We observe intense, broad spectral features associated with photoinduced absorption (PIA) (Supporting Information Figure S8), similar to those previously reported in TA spectra for silver–bismuth-based perovskites and Rudorffites.^32,48^ Figure 2b,c presents a comparison between OPTP and TA kinetics recorded in situ under identical excitation conditions. Importantly, the OPTP photoconductivity signal is proportional to the product of charge-carrier mobility and density, whereas TA probed near the band edge is sensitive only to the total density of photogenerated electrons and holes and is therefore unaffected by changes in their mobility. For Pb_2_SbS_2_I_3_ we observe a clear decoupling of the two entities (Figure 2c): while the OPTP photoconductivity signal collapses in picoseconds, the photoexcited population density reflected by TA persists beyond a nanosecond. Such decoupling therefore directly evidences a rapid loss of charge-carrier mobility in Pb_2_SbS_2_I_3_ rather than a charge-carrier depletion. Crucially, trap-mediated recombination would remove charge carriers from the band edge population entirely, and would be expected to produce correlated decays in both signals on comparable time scales. The discrepancy in the OPTP and TA dynamics for Pb_2_SbS_2_I_3_ therefore unambiguously demonstrates that charge carriers remain present but have become immobile. This TA/OPTP decoupling is qualitatively incompatible with defect trapping and is instead a hallmark of self-trapping into a low-mobility localized state, as has been established for other PIMs.^32^ For Sn_2_SbS_2_I_3_ by contrast, the OPTP and TA signals exhibit broadly similar decay dynamics, both persisting beyond the 1 ns measurement window, consistent with an absence of such ultrafast self-localization. Here, the difference between the decay rates of the OPTP and TA signals may indicate photoconductivity decay is caused by charge-carrier trapping in shallow defect states as discussed further below.^49^ We note that the PIA kinetics of Sn_2_SbS_2_I_3_ and Pb_2_SbS_2_I_3_ are comparable, indicating similar levels of trap-mediated recombination, with the difference in OPTP dynamics arising from their different propensity to induce ultrafast charge localization. Control experiments with alternate excitation wavelengths are found to exclude the possibility of contributions from the mesoporous TiO_2_ scaffold or the Sb_2_S_3_ secondary phase to the observed OPTP dynamics (Supporting Information, Figures S6 and S7). While independently prepared films show slight variations in longer-time trap-mediated recombination, the ultrafast localization dynamics in Pb_2_SbS_2_I_3_ remain unchanged across preparations (Supporting Note 3).

The observed differences in charge localization between the two materials are also evident from the way the dynamics change with the initially injected charge-carrier density. We examined how the OPTP response depends on excitation fluence, and found that while Pb_2_SbS_2_I_3_ shows fluence-independent decay dynamics typical for charge localization, Sn_2_SbS_2_I_3_ exhibits the fluence-dependent bimolecular recombination characteristics of free electrons with holes. Figure 3a presents OPTP data for Pb_2_SbS_2_I_3_ as a function of excitation fluence, which show no dependence of the decay dynamics on the initially injected charge-carrier density across the measured range. A fast decay alone, could in principle, be caused by other processes such as exciton formation,^50^ Auger processes, or intervalley scattering. However, such mechanisms would depend on excitation fluence, while the observed fluence-independent loss of photoconductivity on a picosecond time scale is a well-established signature of ultrafast charge-carrier localization in PIM systems.^39,40^ To gain further quantitative insight into the dynamics of charge-carrier localization, we fit the transients to the two-level localization model (Supporting Information Note 4) developed by Wright et al.,^29^ as depicted in the inset of Figure 3a. In this model, the initially photoexcited charge-carrier density, *n*_deloc_, populates a delocalized state with a higher mobility, μ_deloc_, thereby determining the initial OPTP signal amplitude at *t*_0_. These charge carriers are rapidly transferred to a localized state of lower mobility, μ_loc_, at a rate *k*_loc_. For the localized state, the remnant charge-carrier density *n*_loc_ recombines via a monomolecular process with rate *k*_1_ to the ground state. The excellent fit between this model and the OPTP data for Pb_2_SbS_2_I_3_ confirms that for this mixed-metal chalcohalide, ultrafast charge localization is indeed the dominant mechanism.

**Figure 3:**
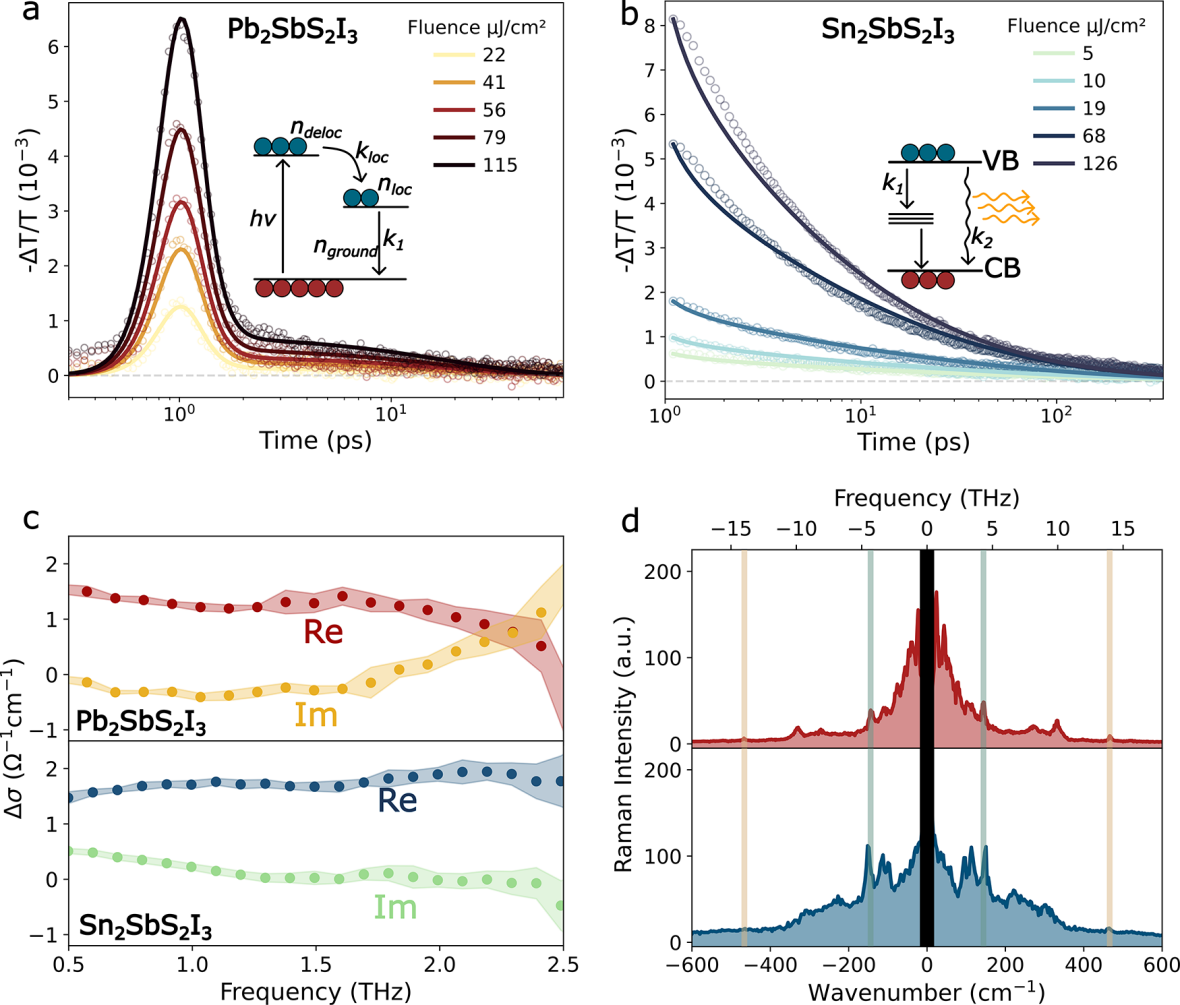
**| Fluence dependent charge-carrier dynamics and vibrational properties of Pb**_2_ **SbS**_2_ **I**_3_ **and Sn**_2_ **SbS**_2_ **I**_3_ **thin films.** (a-b) OPTP transients of Pb_2_SbS_2_I_3_ and Sn_2_SbS_2_I_3_ thin films measured at varying excitation fluences, globally fitted using the corresponding models (solid lines) discussed in the text. Schematic representations of the models are shown. For both panels, the time of excitation *t*_0_ was chosen at 1 ps to allow visualization on a logarithmic time scale. (c) Real and imaginary components of the photoinduced THz conductivity spectra for both materials, taken 0.5 ps after the OPTP onset. Solid points represent experimental data while the surrounding regions denote the experimental uncertainty. (d) Ultralow-frequency Raman spectra. Green and brown lines represent phonon responses from the substrate i.e. TiO_2_ and z-cut quartz, respectively. The Rayleigh scattering peak is masked with a black line.

The reported localization dynamics in related PIMs provide a useful benchmark to assess the severity of localization in Pb_2_SbS_2_I_3_. In the prototypical PIM Cs_2_AgBiBr_6_, localization occurs with a temperature-independent rate of *k*_*loc*_ = 1 ps^–1^ and is accompanied by a drop in mobility by a factor of 3 at room temperature.^29^ Mixed-halide (AgI)_*x*_(BiI_3_)_*y*_ compounds have been shown to exhibit even faster localization dynamics, with *k*_*loc*_ values in the range of 5–9 ps^–1^ and monomolecular recombination rates, *k*_1_ between 0.01 and 0.07 ps^–1^.^32^ Cu_2_AgBiI_6_ similarly displays a barrier-free localization with *k*_*loc*_ = 0.65 ps^–1^, primarily attributed to its reduced dimensionality.^39^ In the related family Cu_4*x*_(AgBi)_1–*x*_I_4_ of systems, reported *k*_*loc*_ rates span 0–5 ps^–1^ and a *k*_1_ between 10^8^ and 10^10^ s^–1^ depending on composition.^41^ By comparison, for Pb_2_SbS_2_I_3_ we extract a particularly fast localization rate of *k*_*loc*_ = 4.53 (±0.3) ps^–1^_,_ consistent with the observed drop in photoconductivity, placing it at the rapid end of the localization spectrum for PIM-type materials. Meanwhile, the electron–hole THz mobility for Pb_2_SbS_2_I_3_ is initially high for the delocalized state (μ_*deloc*_ = 4.6 (±0.2) cm^2^/(V s)) but subsequently declines to only μ_*loc*_ = 0.18 (±0.01) cm^2^/(V s) upon localization—a reduction by a factor of ∼26 within only a few picoseconds. This magnitude in relative mobility decline is substantially larger than those typically observed at room temperature in other PIM systems, with the picosecond collapse in photoconductivity placing Pb_2_SbS_2_I_3_ among the most rapidly localizing PIM absorbers reported to date.^29,33,42^

In contrast, Sn_2_SbS_2_I_3_ exhibits a clear fluence dependence in its decay kinetics, with behavior characteristic of a significant contribution from bimolecular electron–hole recombination rate to the conductivity decay (Figure 3b). Such dynamics are typically described by the *k*_1_ *k*_2_ model capturing first- and second-order recombination in a semiconductor:

$$\frac{dn}{dt}=-k_{1}n-k_{2}n^{2}$$ 1

where *k*_1_ accounts for the trap-mediated monomolecular recombination process, and *k*_2_ is the bimolecular recombination rate of electrons with holes.^49^ Here, to account for the highly dispersive nature of trap-mediated recombination in this disordered semiconductor, as already proposed for other PIMs and chalcohalides,^29^ the Kohlrausch–Williams–Watts^51^ recombination rate can be expressed as *k*_1_ = *k*_*KWW*_ = βτ^–β^ *t* ^β–1^, where τ is the characteristic time constant, and (0 ≤ β ≤ 1) reflects the degree of dispersiveness. The small residual Δ*T/T* signal observed at long delay times (up to 1.2 ns) is therefore attributed to the slowing of the average recombination rate reflecting the dispersive nature of trapping in these materials. Such energetic inhomogeneity is also evident from the broad absorption spectra (Figure 1d) which may result from a distribution of band gaps in the material, and is further supported by the presence of the Sb_2_S_3_ secondary phase (Figure 1c), as previously noted by both Nie et al.^22^ and Manna et al.^43^ We use solutions to eq 1 to perform global fits across the fluence-dependent OPTP data set, and obtain a bimolecular recombination rate constant of *k*_2_ = 1.7 (±0.1) × 10^–9^ cm^3^/s, a typical magnitude for a direct semiconductor,^49^ and an average monomolecular lifetime of τ_*avg*_ = 160 (±20) ps (Supporting Information Note 4). From a linear fit of the maximum Δ*T/T* amplitude against the charge-carrier density (Supporting Information Figure S5) we further extract a value for the electron–hole sum mobility of μ = 2.51 (±0.1) cm^2^/(V s) for the Sn_2_SbS_2_I_3_ film.

We further examine the frequency-dependent photoconductivity spectra (Figure 3c) to explore whether they exhibit signatures of charge-carrier localization. For Sn_2_SbS_2_I_3_, the real component Re­[σ­(ω)] (where ω denotes the THz angular frequency and σ is the photoconductivity) is near flat and positive, and the imaginary Im­[σ­(ω)] remains close to zero across the measured frequency range. Within the Drude model, such a response is characteristic of free-charge transport with short scattering times (ωτ_m_ ≪ 1, where τ_m_ is the momentum-scattering time of charge carriers). In contrast, the photoconductivity spectra recorded for Pb_2_SbS_2_I_3_ thin films shows a qualitatively different signature: both Re­[σ­(ω)] and Im­[σ­(ω)] are flat at low frequencies but cross at ∼2.25 THz. Such a dispersive non-Drude response could potentially arise from specific electron–phonon coupling and charge localization, as previously discussed.^33,40^ On the other hand, the purely Drude-like response observed for Sn_2_SbS_2_I_3_ thin films supports the absence of such localization in these materials. To quantify these differences, we evaluate the Drude factor developed by Milot et al.^52^ for which a value of 1 represents an ideal free-charge response according to the Drude model. We obtained values of 0.90 and 0.77 for Sn_2_SbS_2_I_3_ and Pb_2_SbS_2_I_3_, respectively, thus further supporting our interpretation.

In addition, we probe whether such differences in the propensity to exhibit ultrafast charge localization may arise from variations in lattice anharmonicity. For this purpose, we measured ultralow frequency Raman spectra for both mixed-metal chalcohalides thin films, as shown in Figure 3d. We find that both Sn_2_SbS_2_I_3_ and Pb_2_SbS_2_I_3_ exhibit a so-called “central Raman peak” of increasing amplitude extending toward zero frequency. Similar signals have been observed for a wide range of perovskites and PIMs and have been attributed to lattice anharmonicity and dynamically disordered lattice motion.^47,53,54^ While such lattice softness is a prerequisite for strong electron–phonon coupling and self-trapping, we find here that the emergence of a “central Raman peak” is not a sufficient predictor of the ultrafast localization phenomenon, given that both Sn_2_SbS_2_I_3_ and Pb_2_SbS_2_I_3_ display this peak, but only the Pb analogue exhibits pronounced localization. This conclusion is in agreement with an observation of central Raman peaks in lead halide perovskites, which also do not display ultrafast localization,^53,54^ but rather show long-lived stable conductivity^55^ similar to Sn_2_SbS_2_I_3_.

In order to rationalize these differences between the transport properties of Sn_2_SbS_2_I_3_ and Pb_2_SbS_2_I_3_ we turn toward the structural analysis of these materials. As discussed prior, Sn_2_SbS_2_I_3_ crystallizes in the higher-symmetry orthorhombic *Cmcm* phase, which generally increases the number and equivalence of orbital–orbital overlap pathways. Comparatively, Pb_2_SbS_2_I_3_ crystallizes in the lower-symmetry *P2*_1_ */c* space-group, in which the symmetry limits the equivalence of the orbital-overlap pathways and tends to produce flatter bands. Such reduced crystal symmetry will also lower electronic dimensionality, which has been shown to lower the potential barriers toward charge localization.^38,40,56,57^ Consistent with this picture, recent A_2_BCh_2_X_3_ design-rule calculations suggest larger electron effective masses in lower-symmetry *P2*_1_ */c* phases compared with *Cmcm*/*Cmc2*_1_ polymorphs, supporting reduced electronic connectivity and an enhanced tendency for localization.^26^ Further, Sn_2_SbS_2_I_3_ (*Cmcm*) contains favorable orbital contributions to its band-edges: the Sn (s) lone-pair hybridizes with the S/I (p) orbitals, promoting antibonding band-edge states and enhanced polarizability.^13,21,26^ Note that cation chemistry and lattice symmetry are causally linked in their influence on self-localization. The substitution of Pb^2+^ with Sn^2+^ drives the transition from the monoclinic *P*2_1_/*c* to orthorhombic *Cmcm*, as established by the documented crystal chemistry of this material family^21,26^ and supported by our XRD and Rietveld refinement data. We therefore attribute the absence of ultrafast localization in Sn_2_SbS_2_I_3_ primarily to its higher-symmetry lattice, whether described as the average *Cmcm* phase or as locally polar *Cmc2*_1_ domains.^25^ In either case, the structure remains higher in symmetry than *P2*_*1*_ */c* Pb_2_SbS_2_I_3_, providing greater electronic connectivity and hence a higher barrier to self-trapping. The theoretical framework underpinning this localization behavior and the role of electronic dimensionality in modulating self-trapping barriers is discussed in detail in Buizza et al., 2021.^40^ Conversely, the lower-symmetry *P*2_1_/*c* lattice of Pb_2_SbS_2_I_3_ reduces the electronic connectivity, lowering the self-trapping barrier and driving localization into low-mobility states within a few picoseconds. Differences in band-edge orbital character and local polar fluctuations may further modulate electron–phonon coupling, but the dominant trend observed here correlates with symmetry-controlled electronic dimensionality.^40^ It is worth noting that the initial mobility for Sn_2_SbS_2_I_3_ is lower than that of the delocalized Pb_2_SbS_2_I_3_ state; this can be understood by comparison of the local distortion index extracted from Rietveld refinement (*D* = 4.9% for Pb_2_SbS_2_I_3_ vs *D* = 10.1% for Sn_2_SbS_2_I_3_), which indicates a more perturbed local environment that enhances scattering and reduces momentum relaxation time. In addition, the presence of a minor Sb_2_S_3_ phase may introduce further disorder or interfacial scattering pathways, contributing to the reduced mobility. Despite this, the higher global symmetry of the *Cmcm* framework preserves the electronic connectivity and suppresses ultrafast localization, highlighting that local structural disorder limits mobility but does not necessarily drive self-trapping.

## Conclusions

In this work, we establish a practical design criterion for mitigating ultrafast charge-carrier localization in PIMs through a combined structural and spectroscopic study of Pb_2_SbS_2_I_3_ and Sn_2_SbS_2_I_3_. Despite their chemical similarity, these materials display qualitatively different photoconductivity: upon tuning the metal cation from Pb to Sn, a rapid picosecond localization that collapses the photoconductivity is replaced by a sustained photoconductivity, without the signatures of ultrafast self-trapping. Our results suggest that these contrasting behaviors correlate strongly with differences in lattice symmetry and electronic connectivity. Sn_2_SbS_2_I_3_ adopts a higher-symmetry *Cmcm* framework on average, associated with a more electronically connected structure, whereas Pb_2_SbS_2_I_3_ crystallizes in a lower-symmetry *P*2_1_/*c* lattice. Ultrafast measurements further indicate that these symmetry differences coincide with distinct propensities for phonon-mediated charge-carrier localization. In Pb_2_SbS_2_I_3_, the photoconductivity decay is fluence-independent on picosecond time scales and decoupled from the charge-carrier population dynamics measured by transient absorption, consistent with a rapid collapse into a localized, low-mobility state. This interpretation is supported by the non-Drude-like photoconductivity spectrum, indicative of strong coupling of charge carriers to the underlying lattice. By contrast, Sn_2_SbS_2_I_3_ exhibits a predominantly Drude-like conductivity response and decay dynamics well described by a conventional recombination model, consistent with the absence of ultrafast self-trapping. Together, these findings demonstrate that tuning of cation chemistry can be utilized to increase lattice symmetry and suppress intrinsic localization in chalcohalide absorbers, offering a transferable design principle for PIMs with effective long-range transport. Further work may optimize film processing conditions to reduce trap-mediated losses in Sn_2_SbS_2_I_3_, and examine the role of electron–phonon coupling contributions, including Fröhlich interactions and acoustic deformation potentials, on self-localization in the mixed-metal chalcohalide family.

## Supplementary Material



## Notes

Film fabrication and measurement methods; Rietveld refinement parameters and atomic positions for Pb_2_SbS_2_I_3_ and Sn_2_SbS_2_I_3_; extraction of THz mobility; additional ultrafast spectroscopy data including excitation wavelength and geometry control experiments, transient absorption spectra, and reproducibility measurements; fitting procedures for OPTP kinetics including the standard second-order recombination model and two-level localization model; scanning electron microscopy images; ultralow-frequency Raman reference spectrum. (PDF)
