# Supplementary material for: Overcoming Charge-Carrier Localization in Metal Chalcohalides
Source: J Am Chem Soc. 2026 Jun 22;148(25):26116–24. doi: 10.1021/jacs.6c05142 (PMC13339132; doi:10.1021/jacs.6c05142)
Supplement: Supplementary file 1 [file ja6c05142_si_001.pdf]

Supporting Information: Overcoming Charge-Carrier Localisation in Metal Chalcohalides

*Bembe C. Mackintosh[1], Marcello Righetto[1,2], G. Krishnamurthy Grandhi[3], Thomas B. Haward[1], Noolu Srinivasa Manikanta Viswanath[4], Joshua R. S. Lilly[1], Siyu Yan[1], Jae Eun Lee[1], Snigdha Lal[1], Alan R. Bowman[1], Michael B. Johnston[1], Paola Vivo[3], Laura M. Herz [1]**

[1.] Department of Physics, University of Oxford, Clarendon Laboratory, Parks Road, Oxford, OX1 3PU, United Kingdom

[2.] Department of Chemical Science, Università degli Studi di Padova, Via Marzolo 1, I-35131 Padova, Italy

[3.] Hybrid Solar Cells, Faculty of Engineering and Natural Sciences, Tampere University, P.O. Box 541, FI-33014 Tampere, Finland

[4.] Division of Materials Science and Engineering, Hanyang University, 222 Wangsimni-ro, Seongdong-gu, Seoul 04763, Republic of Korea

*Corresponding Author's Email: laura.herz@physics.ox.ac.uk

Keywords: Lead-Free Semiconductors, Ultrafast Localisation, THz Spectroscopy, Charge-Carrier transport, Photovoltaic Materials, Chalcohalides, Perovskite-Inspired Materials

Table of Contents

1. Film fabrication *3*

Materials 3

Substrate cleaning and mesoporous layer preparation 3

$Sn_2SbS_2I_3$ precursor and film formation on mp-TiO_2 3

$Pb_2SbS_2I_3$ precursor and film formation on mp-TiO_2 4

2. Measurement and Analysis Methods *5*

X-Ray Diffraction 5

Rietveld Refinement 5

Absorption Measurements 6

Photoluminescence Measurements 6

Optical-Pump Terahertz-Probe Spectroscopy 6

Transient Absorption Spectroscopy 7

Ultralow Frequency Raman Spectroscopy 8

3. Scanning Electron Microscopy *9*

4. Supporting Note 1: Rietveld Refinement *10*

Rietveld Refinement of $Pb_2SbS_2I_3$ 10

Rietveld Refinement of $Sn_2SbS_2I_3$ 14

Comparison of Rietveld Refinement Parameters 17

5. Supporting Note 2: Extraction of THz Mobility *18*

6. Supporting Note 3: Additional Ultrafast Spectroscopy Data (TA & OPTP) *21*

7. Supporting Note 4: Fitting OPTP Kinetics *25*

Standard Second-Order Recombination Model 25

Charge-Carrier Localization: Two-Level Mobility Model 27

8. Supporting Note 5: Ultralow Frequency Raman *29*

9. References *31*

1. Film Fabrication

Materials

Z-cut quartz substrates (13 mm diameter, 2 mm thickness; UQG Optics) were used. N,N-dimethylformamide (DMF, anhydrous, 99.8%, Sigma-Aldrich), acetone (99.8%, Sigma-Aldrich), isopropyl alcohol (IPA, anhydrous, 99.8%, Sigma-Aldrich), ethanol (99.5%, Thermo Fisher Scientific), and deionized water were used as solvents. Tin(II) iodide (SnI_2, ultra dry, 99.999%, Thermo Scientific/Alfa Aesar), antimony(III) chloride ($SbCl_3$, 99.99%, Thermo Scientific), thiourea (≥99.0%, Sigma-Aldrich), and lead(II) iodide (PbI_2, ≥98%, TCI) were used without further purification. The mesoscopic scaffold was prepared from 30 NR-D Titania Paste (Greatcell Solar Materials). All solution handling and film formation steps were performed in a nitrogen-filled glovebox unless noted otherwise. Neither $Sn_2SbS_2I_3$ nor $Pb_2SbS_2I_3$ formed as films on bare glass or quartz; reproducible chalcohalide formation required the mesoporous TiO_2 scaffold.

Substrate cleaning and mesoporous layer preparation

Quartz substrates were cleaned in an ultrasonic sequence of aqueous Mucasol (2% v/v in water), deionized water, acetone, and IPA (5 min each at room temperature). Substrates were dried under a nitrogen stream and treated in a UV–ozone cleaner for 15 min. The TiO_2 paste was diluted in ethanol to ~310 mg mL^{-1}, then spin-coated at 4000 rpm for 30 s and annealed at 450 °C in air for 30 min to form the mesoporous TiO_2 (mp-TiO_2) layer.

$Sn_2SbS_2I_3$ precursor and film formation on mp-TiO_2

$Sn_2SbS_2I_3$ film preparation followed a literature-inspired route with slight modifications.[1,2] The precursor solution was prepared by dissolving SnI_2 (260 mg), $SbCl_3$ (228 mg), and thiourea (137 mg) in a 0.7:1.0:1.8 molar ratio in anhydrous DMF (1.00 mL) at room temperature with stirring until clear. The solution was deposited onto the mp-TiO_2-coated quartz and spin-coated

at 1000 rpm for 60 s in a single step. A two-step annealing protocol was then employed: films were first heated directly to 180 °C (color change from yellow to orange), and subsequently heated to 300 °C for 5 min, after which the film appeared black.

$Pb_2SbS_2I_3$ precursor and film formation on mp-TiO_2

The preparation of $Pb_2SbS_2I_3$ films was carried out in a manner similar to that of $Sn_2SbS_2I_3$ films. The $Pb_2SbS_2I_3$ precursor solution was prepared by dissolving PbI_2 (323 mg), $SbCl_3$ (228 mg), and thiourea (137 mg) in a 0.7:1.0:1.8 molar ratio in anhydrous DMF (1.00 mL) at room temperature with stirring until clear (dissolution typically required 2–3 h). The solution (100 μL per substrate) was dispensed onto mp-TiO_2-coated quartz and spin-coated at 1000 rpm for 45 s in a single step. Films were crystallized using a two-step hotplate anneal: 170 °C for 5–10 min, then 300 °C for 5 min.

2. Measurement and Analysis Methods

X-Ray Diffraction

The X-ray diffraction patterns for the $Pb_2SbS_2I_3$ and $Sn_2SbS_2I_3$ films were acquired via Panalytical X'Pert and Panalytical Empyrean diffractometers respectively. In both cases, the Cu-Kα_1 line was utilised. A substrate reference peak (z-cut quartz) was utilised to correct for any angular tilt.

Rietveld Refinement

The Rietveld refinement methodology was employed to analyse the X-ray diffraction (PXRD) data of the $Sn_2SbS_2I_3$ and $Pb_2SbS_2I_3$ samples, utilizing the General Structure Analysis System (GSAS) software for data collection and processing. The refinement commenced with the reported crystal structures of the parent phases serving as initial models, facilitating an iterative least-squares fitting process aimed at optimizing several key parameters, including lattice parameters, atomic positions, site occupancies, and thermal displacement parameters. A pseudo-Voigt profile function was used to model the peak shapes in the diffraction pattern, while background intensities were fitted with a polynomial function to account for instrumental noise and scattering contributions. Convergence of the refinement was evaluated using various R-factor metrics, such as the weighted R-factor (R_{wp}) and the profile R-factor (R_p), alongside goodness-of-fit metrics to ensure a reliable correlation between the observed and calculated diffraction patterns. Following the full pattern refinement, bond angles and lengths were extracted from GSAS, leading to the calculation of distortion index values based on the extracted bond lengths.

Absorption Measurements

Visible–NIR absorption spectra were obtained with a Bruker Vertex 80v Fourier-transform infrared (FTIR) spectrometer, employing a transmission/reflection accessory for measurement. Spectra were taken using a tungsten halogen source, and a Si detector.

Photoluminescence Measurements

For measurements of $Pb_2SbS_2I_3$, steady-state photoluminescence (PL) measurements were performed using a grating spectrometer coupled to an intensified charge-coupled device (iCCD) detector. Excitation was provided by a 3.1 eV diode laser (PicoQuant LDH-D-C-398M). The emitted light was spectrally dispersed by the monochromator (Princeton Instruments SP-2558) and detected with a silicon iCCD (Princeton Instruments PI-MAX4).

For PL measurements of $Sn_2SbS_2I_3$, excitation was provided by 80 fs pulses at a 780 nm wavelength generated from a tuneable Ti:Sapphire laser (Mai Tai, Spectra Physics) with 80 MHz repetition rate at an approximate fluence of ~ 17.2 μJ cm^{-2}. The measured PL was detected using a N_2-cooled silicon CCD detector after passing through an 830 nm long-pass filter.

Optical-Pump Terahertz-Probe Spectroscopy

Optical-pump-terahertz-probe (OPTP) measurements were performed using a setup described in detail elsewhere.[3] Briefly, 1.55 eV pulses with 35 fs duration and a 5 kHz repetition rate were generated by an amplified Ti:sapphire laser system (Spectra-Physics Spitfire). THz probe pulses were generated with a spintronic emitter which consists of 1.9 nm Tungsten/2.0 nm $Co_{40}Fe_{40}Be_{20}$/1.9 nm platinum coated with anti-reflectivity and high-reflectivity coatings. Single-cycle THz radiation pulses were produced in the spintronic emitter via the inverse spin Hall effect.[4] The THz and optical pump beams were incident on the thin-film sample in a collinear geometry.

Fractional changes in THz transmission were monitored using free-space electro-optic (EO) sampling. The detection system consisted of a 1 mm-thick ZnTe (110) crystal, a Wollaston prism, and a pair of balanced photodiodes. Films were photoexcited either by 3.1 eV photon-energy pulses obtained by frequency-doubling the fundamental in a beta-barium borate (BBO) crystal, or at 2.34 eV (530 nm), generated by a traveling-wave optical parametric amplifier of super fluorescence (TOPAS) based on the sum-frequency of the signal pulse train. Samples were excited from the top, air-facing side, as opposed to the bottom, quartz facing side, unless stated otherwise (e.g. for the measurements discussed in Supporting Note 3).

During OPTP measurements, the THz generation, detection optics, and samples were maintained under vacuum (< 0.1 mbar).

Transient Absorption Spectroscopy

Transient absorption (TA) measurements were performed simultaneously and in situ with the optical-pump–terahertz-probe (OPTP) experiment, as described before.[5] A broadband optical probe continuum was generated by focusing a fraction of the 800 nm fundamental laser output into a 3 mm-thick c-cut sapphire disk. The probe beam was focused onto the sample, spatially overlapped with both the THz probe and optical pump beams, collected in transmission. The transmitted light was detected using either a Si photodiode for measurements between 2.3 – 1.45 eV (400 – 850 nm) or an InGaAs detector for measurements between 1.46 – 1.12 eV (850 to 1100 nm).

The presence of either probe beam did not influence the measured OPTP or TA dynamics. The optical and THz probe time delays were matched to ensure comparable temporal resolution, and $t = 0$ was manually adjusted for each dataset. The wavelength-dependent variation of $t = 0$

in the TA measurements was corrected to account for the group-velocity dispersion of the optical probe.

Ultralow Frequency Raman Spectroscopy

As described by our group previously,[6,7] a narrowband Ti:sapphire continuous-wave (CW) laser (Spectra Physics Matisse 2 TS), pumped by a 532 nm Spectra Physics Millennia CW source, was used as the Raman excitation at 900 nm. The beam was focused onto the films using a 0.5 NA microscope objective (Olympus LMPLFLN50×), and the scattered light was collected in a backscattering configuration. Raman signals were dispersed by a Horiba iHR320 spectrometer and detected with a Symphony silicon CCD. To suppress Rayleigh scattering and further narrow the excitation line, a set of OptiGrate Bragg filters was placed in the excitation and collection paths.

3. Scanning Electron Microscopy

Cross-sectional scanning electron microscopy (SEM) images were acquired using an FEI Quanta 600 FEG microscope operated at an accelerating voltage of 5 kV, with the beam current defined by a spot size of 3.0.

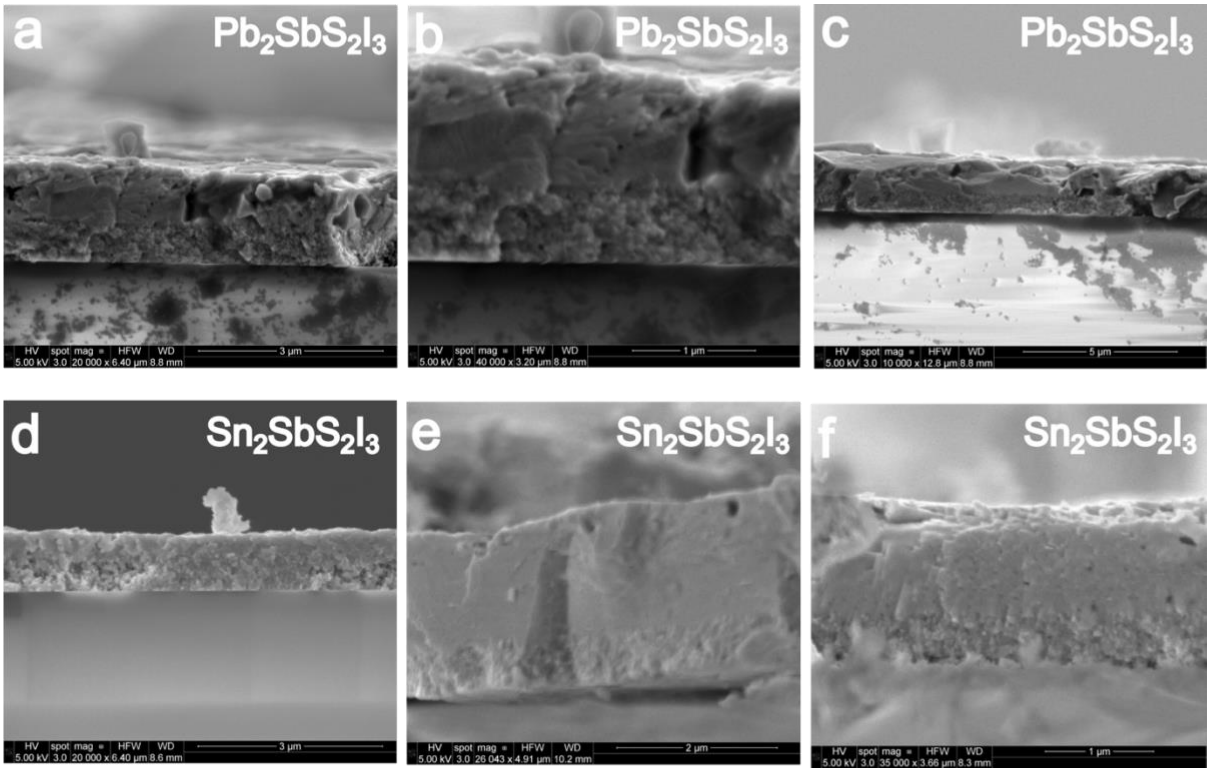

Figure S1| **Cross-sectional SEM images taken of thin-film samples at varying magnifications**. Panels (a-c) show $Pb_2SbS_2I_3$- based films, while (d-f) show $Sn_2SbS_2I_3$ -based films.

Films were imaged through cross-sectional SEM, as shown in Figure S1. For both types of chalcohalides, the compact layer of TiO_2 first deposited on the quartz substrate to aid adhesion is clearly visible, followed by a top layer incorporating mostly the active chalcohalide material. As a result, laser excitation from the front side (i.e. not through the substrate) mostly generates charge carriers in the chalcohalide bulk material, which is the case even if photon wavelengths above the bandgap of TiO_2 are used (see evidence provided further below).

4. Supporting Note 1: Rietveld Refinement

Rietveld Refinement of $Pb_2SbS_2I_3$

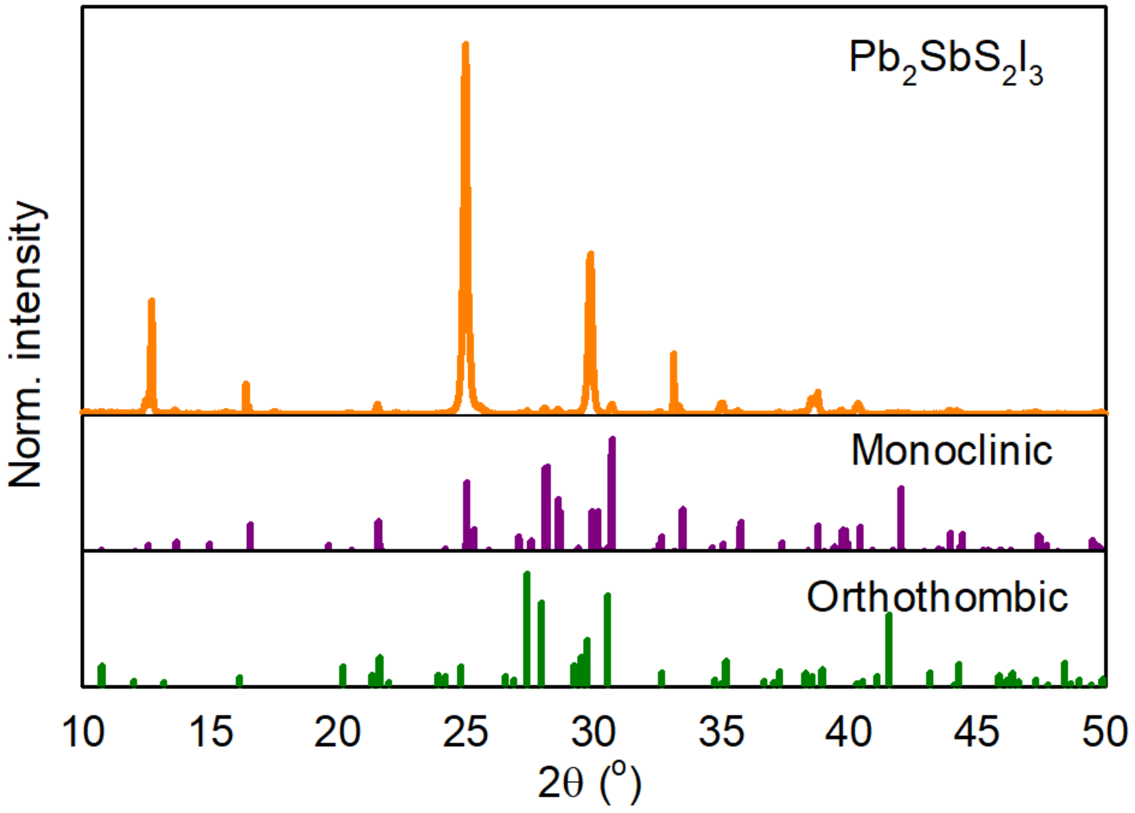

Figure S2 | **Comparison of experimental and reference XRD patterns for $Pb_2SbS_2I_3$.** Experimental XRD pattern (orange) shown alongside the reported monoclinic (purple) and orthorhombic (green) reference patterns for comparison.

X-ray diffraction (XRD) analysis indicates that the Pb–Sb chalcohalide composition adopts a **monoclinic** rather than an orthorhombic crystal structure. As shown in Figure S2 the most intense diffraction peaks observed at 25° and 29.5° closely correspond to the characteristic reflections and relative intensities of the standard monoclinic phase pattern. Accordingly, the monoclinic structural model was used as the basis for Rietveld refinement of the XRD data for $Pb_2SbS_2I_3$.

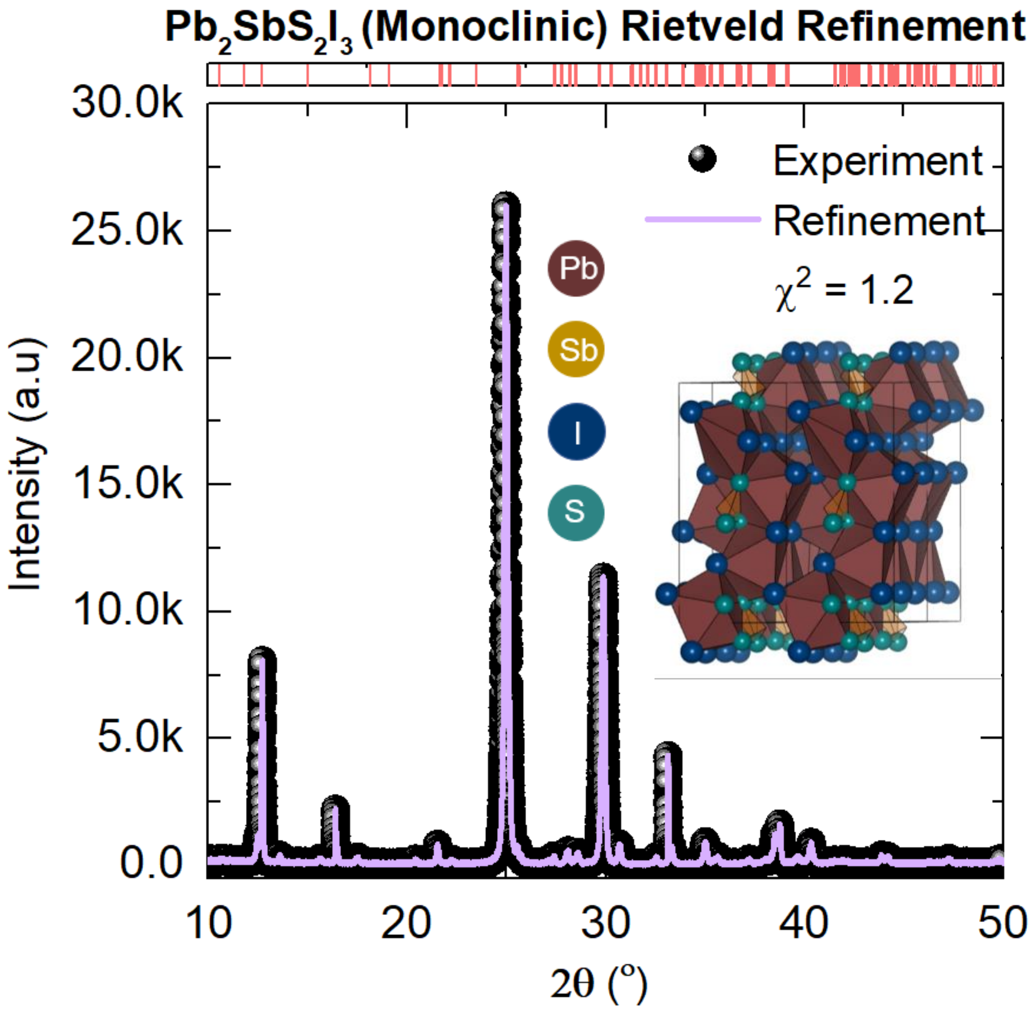

Figure S3 | XRD refinement and structural comparison for $Pb_2SbS_2I_3$. Rietveld refinement (purple) shown alongside the experimental XRD pattern (black), with the simulated crystal structure included in the inset. Tick marks at the top indicate the expected peak positions for the corresponding reference space-group pattern.

Table S1: Rietveld refinement parameters of $Pb_2SbS_2I_3$ obtained from the diffraction data at room temperature. The numbers in parentheses are the estimated standard deviations of the last significant figure.

**Compound**	**$Pb_2SbS_2I_3$**
Space group	$P2_1/c$
a (Å)	9.59(7)
b (Å)	16.70(5)
c (Å)	3.78(3)
V (Å^3)	459.21(5)
R_{wp} (%)	2.73
R_{exp} (%)	1.83
χ^2	1.2

Table S2: Atomic positions of $Pb_2SbS_2I_3$ obtained from the diffraction data at room temperature. The numbers in parentheses are the estimated standard deviations of the last significant figure.

Atom	Wyckoff position	*x*	*y*	*z*	*g*	100xU_{iso}(Å)^2
Pb1	4*e*	0.761(3)	-0.036(7)	0.187(5)	1.00	0.028(3)
Pb2	4*e*	0.647(2)	0.218(3)	0.141(7)	1.00	0.304(12)
Sb	4*e*	0.357(3)	0.063(5)	0.187(7)	1.00	0.37(2)
I1	4*e*	0.866(3)	0.128(8)	-0.317(7)	1.00	0.06(2)
I2	4*e*	0.268(9)	0.270(5)	0.274(3)	1.00	0.02(5)
I3	4*e*	0.873(2)	0.122(5)	0.693(7)	1.00	0.04(2)
S1	4*e*	0.211(2)	0.042(5)	0.472(7)	1.00	0.26 (2)
S2	4*e*	0.739(5)	-0.022(7)	0.403(9)	1.00	0.80(7)

Rietveld Refinement of $Sn_2SbS_2I_3$

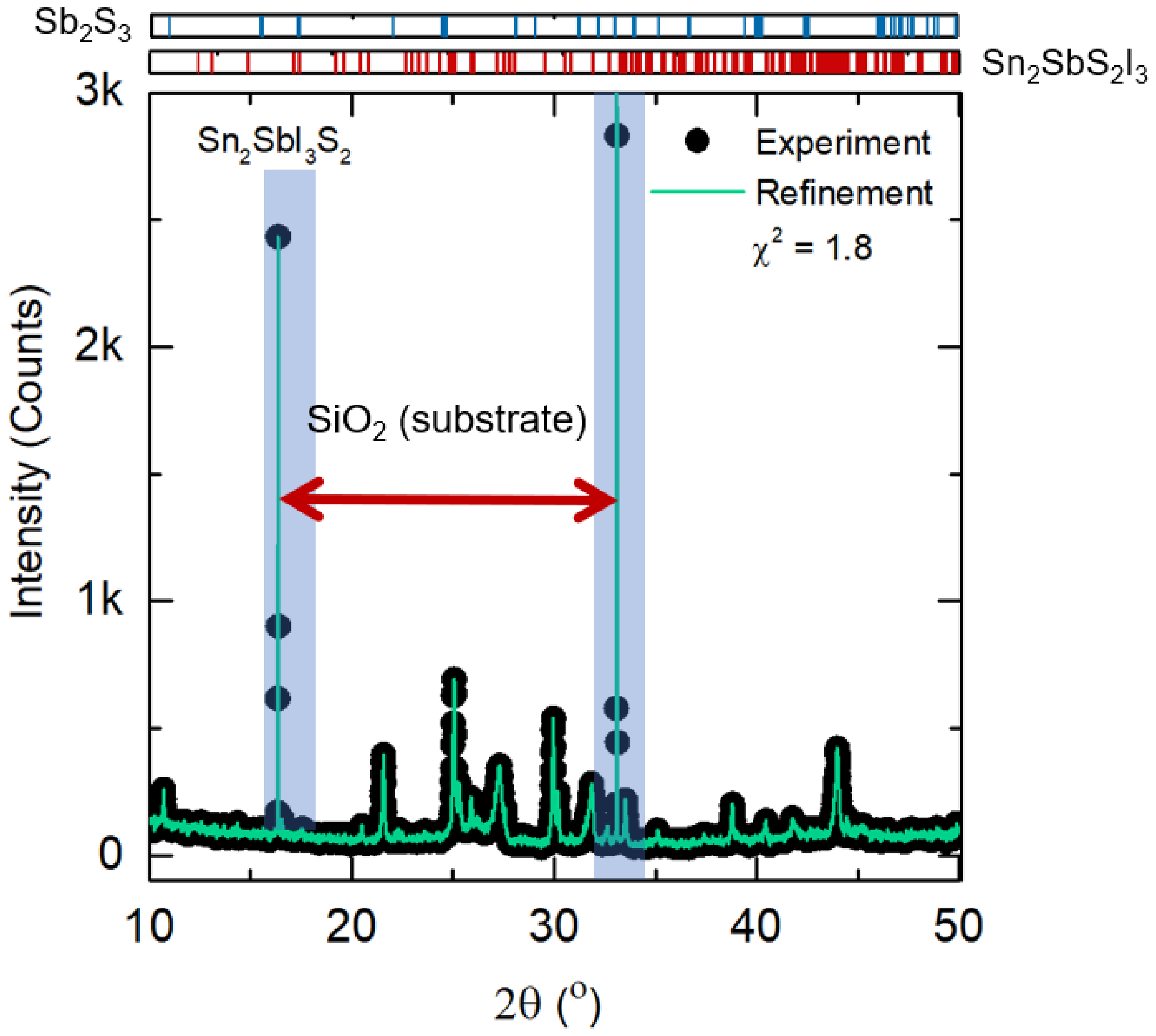

Figure S4 | XRD refinement and phase identification for $Sn_2SbS_2I_3$. Rietveld refinement (green) shown alongside the experimental XRD pattern (black). Reflections arising from the SiO_2 substrate are highlighted in blue. Red and blue tick marks at the top indicate the expected peak positions for both the $Sn_2SbS_2I_3$ and Sb_2S_3 reference patterns respectively.

Table S3: Rietveld refinement parameters of $Sn_2SbS_2I_3$ obtained from the diffraction data at room temperature. The numbers in parentheses are the estimated standard deviations of the last significant figure.

**Compound**	**$Sn_2SbS_2I_3$**
Space group	*Cmcm*
Structure	Orthorhombic
a/Å	4.27(7)
b/Å	14.06(3)
c/Å	16.46(9)
V/Å^3	988.85(5)
R_{wp}	3.8%
χ^2	1.8

Table S4: Atomic positions of $Sn_2SbS_2I_3$ obtained from the diffraction data at room temperature. The numbers in parentheses are the estimated standard deviations of the last significant figure.

**Atom**	**Wyckoff position**	***x***	***y***	***z***	***g***	**100xU_{iso}(Å)^2**
Sn	8*f*	0.00	0.1334(5)	0.0182(7)	1.00	0.039(17)
Sb	4*c*	0.00	0.7985(7)	0.25	1.00	0.090(8)
S	8*f*	0.00	0.7281(3)	0.0790(2)	1.00	0.054(12)
I	8*f*	0.00	0.4407(5)	0.1219(5)	1.00	0.0911(7)
I	4*c*	0.00	0.1444(7)	0.25	1.00	0.0160(4)

Table S5: Extracted cation coordination, dihedral angles and local distortion indices for the polyhedra.

**Composition**	**Coordination**	**D (%)**	**Dihedral angle**
$Pb_2SbS_2I_3$	PbI_4S_3	4.9 (Pb)	123.15
$Sn_2SbS_2I_3$	SnS_3I_2	10.1 (Sn)	84.65

Comparison of Rietveld Refinement Parameters

Rietveld refinement confirms that $Pb_2SbS_2I_3$ crystallises in the monoclinic *P2₁/c* structure, whereas $Sn_2SbS_2I_3$ adopts the orthorhombic *Cmcm* phase. Beyond the space-group assignment, the refinement also reveals notable differences in local geometry: $Sn_2SbS_2I_3$ exhibits a pronounced ~80–90° chain kink and a larger local distortion factor (D = 10.1%) compared with $Pb_2SbS_2I_3$ (D = 4.9%), indicative of a more perturbed coordination environment around the cations. Note that the mesoporous TiO_2 scaffold exhibits low crystallinity, resulting in typically broadened or absent Bragg peaks in the XRD pattern. The Rietveld refinement was thus performed without an explicit TiO_2 phase.

5. Supporting Note 2: Extraction of THz Mobility

This section discusses the extraction of charge-carrier mobility, following the protocol established by Wehrenfennig *et al.*[8] further formalised by Xia *et al.*[9] and used a number of times by our group.[3,5,10–15] The measured fractional change in THz transmission, $\Delta T/T$, was converted to a photoconductivity signal, $\Delta\sigma$, which is related to the photoconductivity, the carrier density, n, and the effective electron-hole sum mobility, μ, through

$$\Delta\sigma = en\mu. \quad (1)$$

In thin films with a strong optical absorption, the photoexcited charge-carrier density varies with depth according to the Beer–Lambert law. This leads to a depth-dependent conductivity profile

$$\Delta\sigma = \Delta\sigma_0 e^{-\alpha z}, \quad (2)$$

where $\Delta\sigma_0$ is the surface photoconductivity and α is the absorption coefficient at the excitation wavelength. If the film thickness is smaller than the optical absorption length, $1/\alpha$, the excitation can be considered uniform; otherwise the excited region is confined to a surface layer with thickness $t \approx 1/\alpha$.

Under the transmission geometry used in our measurements, the observable THz response depends on the depth-averaged photoconductivity. In this regime, the total photoconductivity sensed by the THz probe may be written as

$$\Delta\sigma = -C\left(\frac{\Delta T}{T}\right), \quad (3)$$

where the constant, C, incorporates the refractive-index and boundary condition factors. A full solution to the THz wave equation,[13] for a thin conductive layer with arbitrary $\Delta\sigma(z)$ reduces the problem to the sheet photoconductivity

$$\Delta S = -\varepsilon_0 c(n_A + n_B)\left(\frac{\Delta T}{T}\right), \tag{4}$$

with

$$\Delta S = \int_0^d \Delta\sigma(z)dz\,, \tag{5}$$

and n_A (= 1) and n_B (= 2.13) the refractive indices at THz frequencies on either side of the film.

To convert ΔS into a mobility value, the number of photogenerated charges N must be determined. This quantity is proportional to the number of absorbed pump photons and is expressed as

$$N = \phi \frac{E}{\left(\frac{hc}{\lambda}\right)}(1 - R - T), \tag{6}$$

where E is the pulse energy, hc/λ is the photon energy, R and T are the pump reflectance and transmission of the film, and ϕ denotes the fraction of the absorbed photons that generate free carriers.

The effective electron-hole sum mobility, which accounts for the unknown branching ratio ϕ, is then obtained by combining the above expressions:

$$\phi\mu = \frac{\Delta S A_{\text{eff}}}{Ne} = -\varepsilon_0 c(n_A + n_B)\frac{hcA_{\text{eff}}}{eE\lambda}\frac{\frac{\Delta T}{T}}{(1 - R - T)}. \tag{7}$$

The factor A_{eff} represents the overlap between the optical pump and THz probe beams, and for Gaussian spatial profiles, is given by $A_{\text{eff}} = 2\pi\left(\sigma_{pump}^2 + \sigma_{THz}^2\right)$, following Xia et al.[9] In absence of any evidence for bound excitonic states in the optical spectra, we assume that $\phi \approx 1$, meaning that the recovered mobility corresponds to the combined electron–hole mobility.

Assuming a lack of recombination processes within the instrument response the effective mobility can be recovered from the OPTP measurement near t=0, by using the above equation, as shown in Figure S5. Here, the points are the maximum $\Delta T/T$ value taken from the OPTP onset, and the line is a linear fit forced through zero.

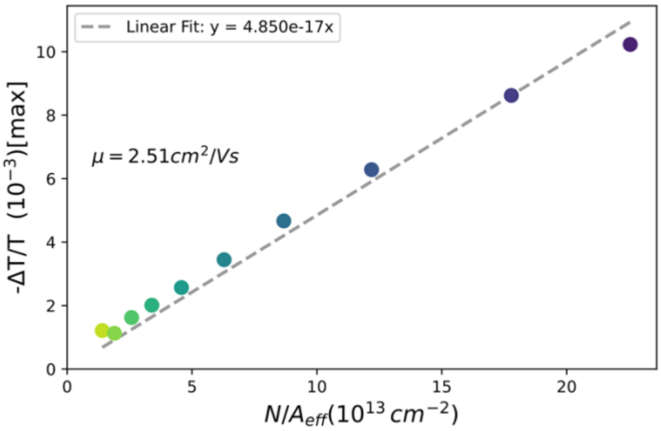

Figure S5 | Extraction of the effective mobility from fluence-dependent OPTP measurements of $Sn_2SbS_2I_3$ films. The initial photoconductivity depends linearly on the photogenerated carrier density N/A_{eff}. Data points represent the experimentally obtained values across different excitation fluences, and the grey line shows the linear fit used to determine the effective electron-hole sum mobility. The extracted mobility is μ = 2.51 cm^2/Vs.

6. Supporting Note 3: Additional Ultrafast Spectroscopy Data (TA & OPTP)

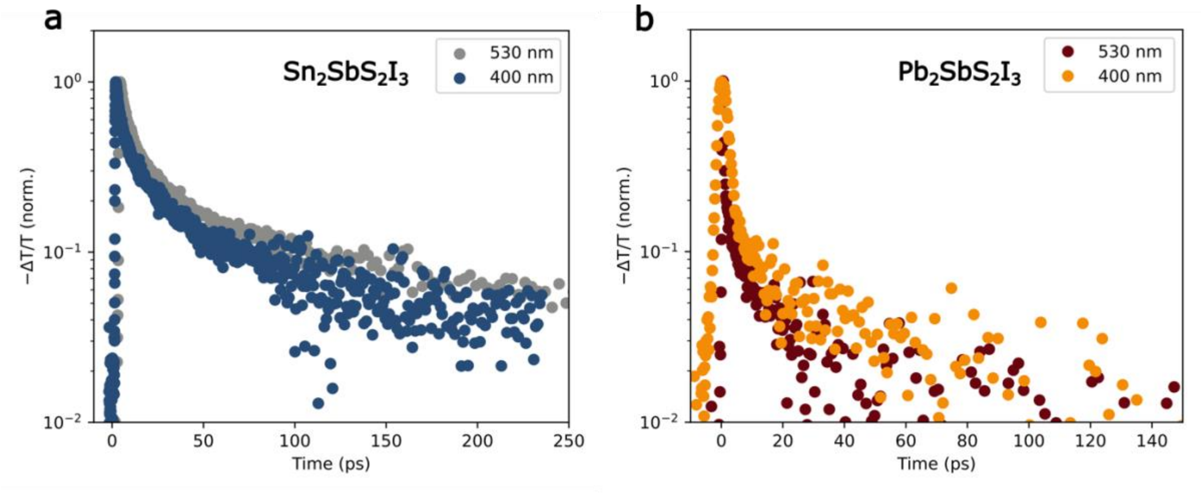

Figure S6 | **OPTP decay kinetics of (a) $Sn_2SbS_2I_3$ and (b) $Pb_2SbS_2I_3$ recorded for excitation with different photon energies, from the surface side**. OPTP photoconductivity transients probed at 400 nm (3.1 eV) and 530 nm (2.3 eV), recorded under excitation fluences of 56 and 64 μJ/cm^2 respectively.

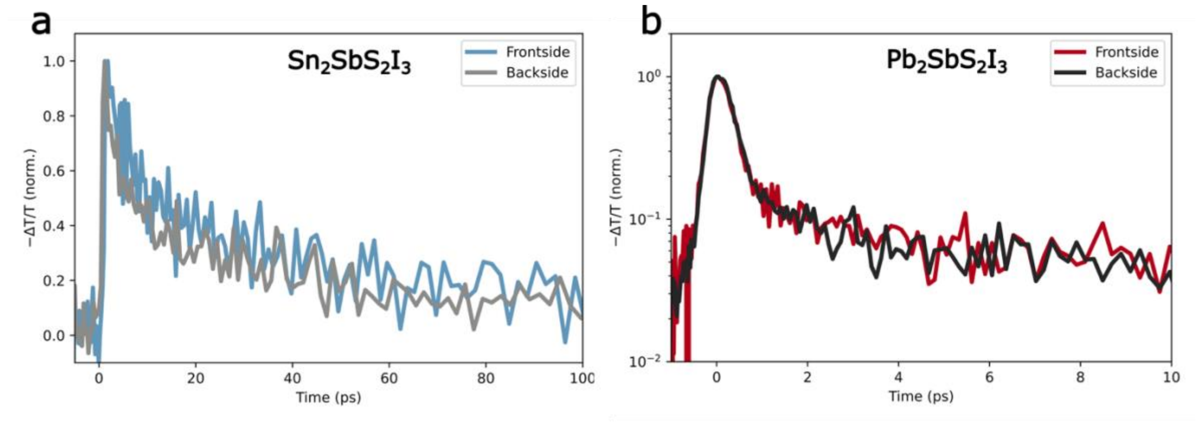

Figure S7 | **OPTP decay kinetics of (a) $Sn_2SbS_2I_3$ and (b) $Pb_2SbS_2I_3$ recorded for different excitation geometries.** OPTP photoconductivity transients recorded under back-side (quartz-first) and front-side (sample-first) optical excitation, corresponding to excitation incident from the substrate and film surface side, respectively. Measurements were performed under an excitation fluence of 40 μJ/cm².

To further establish that the observed OPTP dynamics originate from the mixed-metal chalcohalide rather than from the mesoporous TiO_2 scaffold or from charge-transfer processes at the interface between the two materials, we performed a series of control experiments varying both the excitation wavelength and excitation geometry. As shown in Figure S6, the transient dynamics are largely independent of the excitation wavelength. Excitation at 530 nm, which lies far below the TiO_2 absorption edge[16–18] but remains above the bandgap of the chalcohalide samples, yields dynamics indistinguishable from those obtained under 400 nm excitation. The absence of any measurable change indicates that direct photoexcitation of TiO_2 does not contribute significantly to the observed recombination dynamics.

Additional insight is obtained by comparing excitation from the front (air-facing) and back (quartz-facing) sides of the samples, as shown in Figure S7. Despite the asymmetric sample geometry, as seen in Figure S1, for which there is a higher TiO_2 concentration near the quartz substrate, no discernible differences in the OPTP dynamics are observed. If interfacial charge transfer into TiO_2 were contributing to the measured signal, excitation from the substrate side, where photoexcitation occurs closer to regions of higher TiO_2 density, would be expected to modify the temporal evolution of the response.[19] Such observed invariance thus suggests against a dominant role of TiO_2-related or interfacial charge-transfer processes.

Further evidence is provided by the absence of any fluence dependence in the OPTP dynamics, as shown in Figures 3a and 3b of the main text.

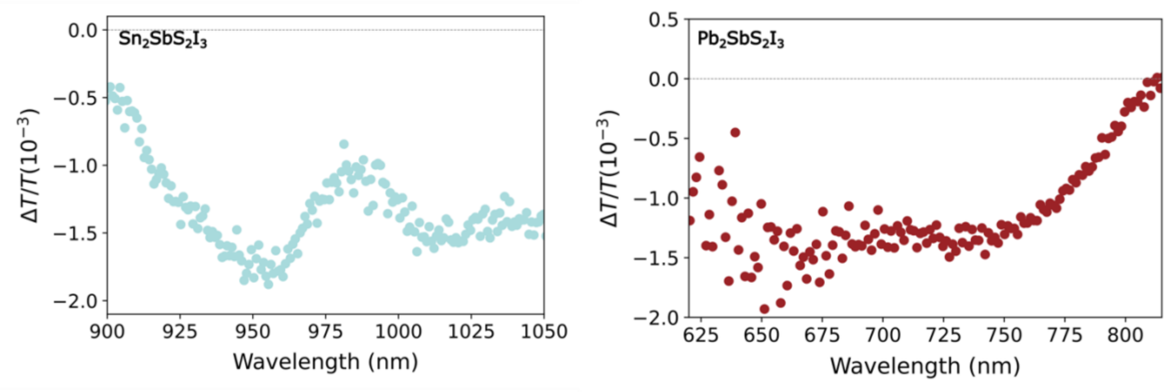

Figure S8 | **Early-time TA spectra of mixed-metal chalcohalide thin films.** Transient absorption spectra at t = 1 ps for $Sn_2SbS_2I_3$ and $Pb_2SbS_2I_3$ thin films excited at fluences of 68 and 53 μJ/cm^2. The spectra capture the immediate photoinduced absorption response associated with the initial carrier distributions.

We note that an analogous argument applies to the Sb_2S_3 secondary phase identified in $Sn_2SbS_2I_3$ by Rietveld refinement (Figure S4). Given that Sb_2S_3 has a larger bandgap than $Sn_2SbS_2I_3$, varying the excitation energy modifies the relative population of charge carriers generated in each phase. Were the observed dynamics to be dominated by the Sb_2S_3 phase then a dependence on excitation energy would be expected. The absence of such effects thus confirms that the OPTP response is intrinsic to the dominant $Sn_2SbS_2I_3$ phase.

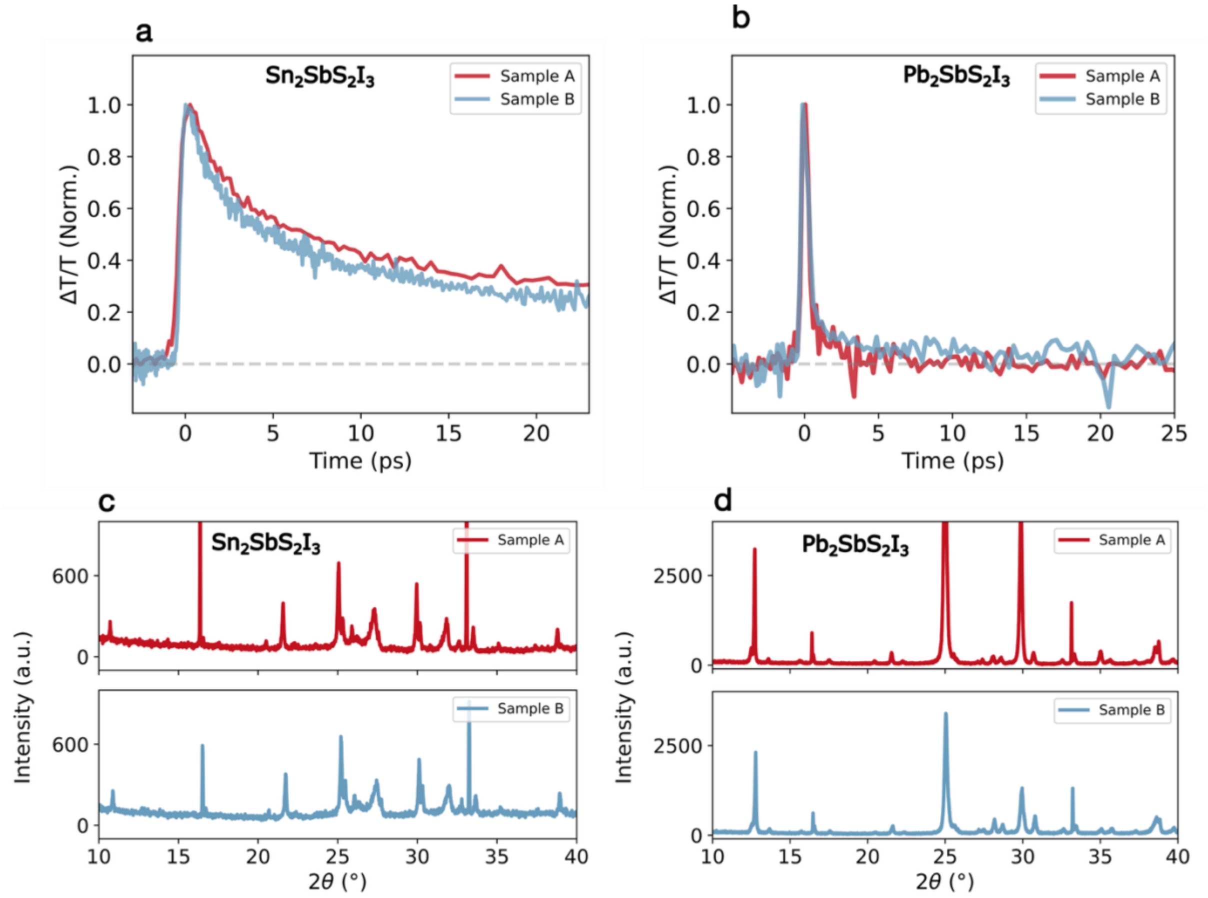

Figure S9 | Control measurements contrasting OPTP kinetics and XRD for two independently prepared films of $Sn_2SbS_2I_3$ and $Pb_2SbS_2I_3$: a) OPTP taken for $Sn_2SbS_2I_3$ film sample A and B, at 19 and 21 μJ/cm^2 respectively. A small difference in the decay rate (dominated by charge-carrier recombination) is observed. b) OPTP taken of $Pb_2SbS_2I_3$ sample A and B, both at 43 μJ/cm^2. No difference can be observed in the initial localisation dynamics apparent in the OPTP decay kinetics, while minor differences are seen in the subsequent decay dominated by charge-carrier recombination. c-d) Matching XRD patterns of $Sn_2SbS_2I_3$ and $Pb_2SbS_2I_3$ film sample A and B.

7. <u>Supporting Note 4: Fitting OPTP Kinetics</u>

Here, we outline the recombination models used to fit the OPTP data based on ordinary differential equations (ODE). These models are represented schematically in Figures 2a, 3a and 3b of the main text.

Standard Second-Order Recombination Model

As briefly described in the manuscript, to quantify the recombination dynamics observed in the OPTP transients of $Sn_2SbS_2I_3$, we model the temporal evolution of the photoconductivity using a rate equation containing both monomolecular and bimolecular contributions. The photoconductivity is proportional to the photoexcited carrier density, *n(t)*, and its decay therefore reflects the net recombination kinetics.

The time dependence of *n(t)* can be described by mechanisms of first and second order recombination, according to the equation

$$\frac{dn}{dt} = -k_1 n(t) - k_2 n(t)^2, \tag{8}$$

where k_1 captures the monomolecular trap-mediated recombination pathways, and k_2 represents the bimolecular electron-hole recombination coefficient.

In systems exhibiting energetic, or structural disorder, as is observed here for $Sn_2SbS_2I_3$, the presence of multiple first-order relaxation pathways leads to the deviation from a single exponential decay. To account for this, the monomolecular term is expressed using a Kohlrausch-Williams-Watts (KWW)[20] stretched exponential function

$$k_1 = k_{KWW} = \beta\tau^{-\beta}t^{\beta-1}, \tag{9}$$

where τ is the characteristic time constant, β $(0 \leq \beta \leq 1)$, reflects the degree of dispersiveness.

This form corresponds to an underlying distribution of recombination rates, rather than a single decay channel, consistent with local energetic variation, thus the parameter τ is not equal to the physical mean molecular lifetime. The appropriate average lifetime, τ_{avg}, is calculated by integrating the corresponding KWW function, yielding

$$\tau_{\mathrm{avg}} = \int_0^\infty \exp\left[-\left(\frac{t}{\tau}\right)^\beta\right] dt = \frac{\tau}{\beta}\Gamma\left(\frac{1}{\beta}\right), \tag{10}$$

where $\Gamma(x)$ is the Gamma function.

To extract the recombination parameters, the OPTP transients were analysed using a global fitting procedure in which all the fluences were modelled simultaneously. To correctly account for the strong optical absorption of the films, the excitation profile was treated explicitly using a depth-resolved approach. The film thickness was divided into a series of thin slices, and each slice at depth z_i was assigned an initial charge-carrier density following a Beer-Lambert distribution, $n_i(0) = n_0 e^{-\alpha z_i}$, where n_0 is the absorbed carrier density at the surface. Each slice was then propagated independently by numerically integrating the rate equation above, and the total photoconductivity at each delay time was obtained by summing the contributions from all slices. Only the initial slice populations varied with fluence, while the recombination parameters $\{\beta, \tau, k_{\mathrm{kww}}, k_2\}$ were constrained to be identical across the full dataset.

Charge-Carrier Localization: Two-Level Mobility Model

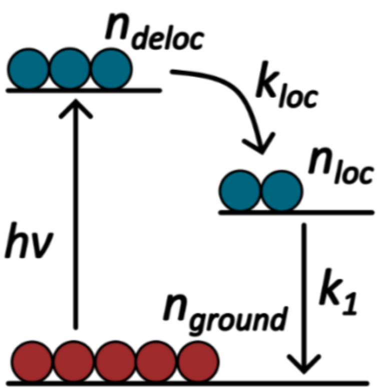

Figure S10 | Schematic illustration of the two-level mobility model. Diagram showing the relaxation of photogenerated carriers from an initial delocalised state with high mobility, μ_{deloc}, to a localised state with reduced mobility, μ_{loc}, along with the associated rate constants governing localisation and recombination, k_{loc} and k_1.

To describe the ultrafast localization dynamics observed in the OPTP transients of $Pb_2SbS_2I_3$, we employ a two-level mobility model similar to that used by Wright *et al.*[10] and Buizza *et al.*[11,12] In this framework, photoexcitation initially generates a population of delocalised charge carriers, $n_{\mathrm{deloc}}(t)$, which possess a high electron–hole sum mobility, μ_{deloc}, as shown in Figure S10. This mobility governs the photoconductivity at the earliest measurement times and represents the transport properties of carriers in delocalised states. Following excitation, these charge-carriers relax into a localised state characterised by a lower mobility, μ_{loc}, and a population $n_{\mathrm{loc}}(t)$.

The localisation process is assumed to be monomolecular in nature and proceeds with a rate constant k_{loc}. Once carriers have relaxed into the localised state, they recombine over longer timescales with a monomolecular recombination rate k_1. The corresponding coupled rate equations are

$$\frac{dn_{\mathrm{deloc}}}{dt} = -k_{\mathrm{loc}} n_{\mathrm{deloc}}, \tag{11}$$

$$\frac{dn_{\mathrm{loc}}}{dt} = k_{\mathrm{loc}} n_{\mathrm{deloc}} - k_1 n_{\mathrm{loc}}, \tag{12}$$

Solving these yields:

$$n_{\mathrm{deloc}}(t) = n_0 e^{-k_{\mathrm{loc}} t}, \tag{13}$$

$$n_{\mathrm{loc}}(t) = n_0 \frac{k_{\mathrm{loc}}}{k_{\mathrm{loc}} - k_1} \left(e^{-k_1 t} - e^{-k_{\mathrm{loc}} t}\right), \tag{14}$$

where n_0 is the total population of the photogenerated charge carriers, calculated from the absorbed pump fluence and the measured absorption coefficient.

Because OPTP conductivity is proportional to the sheet photoconductivity, the total response at time t is given by the mobility-weighted sum of the localised and delocalised populations:

$$\Delta S = \frac{e}{A_{\mathrm{eff}}} \left(n_{\mathrm{deloc}} \mu_{\mathrm{deloc}} + n_{\mathrm{loc}} \mu_{\mathrm{loc}}\right). \tag{15}$$

Combining Equations 4, 13, 14, and 15 gives the analytical expression used to model the OPTP dynamics:

$$\frac{\Delta T}{T} = \frac{-e n_0}{\varepsilon_0 c (n_A + n_B)} \left(\left(\mu_{\mathrm{deloc}} - \frac{\mu_{\mathrm{loc}} k_{\mathrm{loc}}}{k_{\mathrm{loc}} - k_1} \right) e^{-k_{\mathrm{loc}} t} + \left(\frac{\mu_{\mathrm{loc}} k_{\mathrm{loc}}}{k_{\mathrm{loc}} - k_1} \right) e^{-k_1 t} \right). \tag{16}$$

To account for the finite temporal resolution of the OPTP apparatus, this expression is then convoluted with a Gaussian instrument of the width σ_{IRF} (= 200 fs) centred at t_0. The final expression is then used to fit the measured data:

$$\frac{\Delta T}{T}(t-t_0)=\frac{-en_0}{\varepsilon_0 c(n_A+n_B)}\left(\begin{array}{c}\left[\mu_{\text{deloc}}-\frac{\mu_{\text{loc}}k_{\text{loc}}}{k_{\text{loc}}-k_1}\right]e^{-k_{loc}(t-t_0)+\frac{k_{\text{loc}}^2\sigma_{\text{IRF}}^2}{2}}\text{Erfc}\left(\frac{k_{\text{loc}}\sigma_{\text{IRF}}^2-(t-t_0)}{\sqrt{2}\sigma_{\text{IRF}}}\right)+\\ \frac{\mu_{\text{loc}}k_{\text{loc}}}{k_{\text{loc}}-k_1}\,e^{-k_1(t-t_0)+\frac{k_1^2\sigma_{\text{IRF}}^2}{2}}\text{Erfc}\left(\frac{k_1\sigma_{\text{IRF}}^2-(t-t_0)}{\sqrt{2}\sigma_{\text{IRF}}}\right)\end{array}\right),(17)$$

where Erfc(x) is the complementary error function Erfc$(x)=\frac{2}{\sqrt{\pi}}\int_x^\infty e^{-y^2}dy$. The localisation model was then fit globally across the full set of fluence-dependent transients. For each excitation density, only n_0 was allowed to vary, reflecting different numbers of absorbed photons. The intrinsic material parameters, the delocalised and localised mobilities μ_{deloc} and μ_{loc}, and the rate constants k_{loc} and k_1, were constrained to remain identical for all fluences. Here, the extracted parameters are: $\mu_{deloc}=4.6\pm0.2$ cm^2/Vs, $\mu_{loc}=0.18\pm0.01$ cm^2/Vs, $k_{loc}=4.53\pm0.3$ ps^{-1} and $k_1=0.054\pm0.002$ ps^{-1}. Parameter uncertainties were estimated by performing a residual bootstrapping of the global fit (500 resampled refits).

8. Supporting Note 5: Ultralow Frequency Raman

Figure S11 shows an ultralow-frequency Raman spectrum of the bare quartz substrate. The narrow Rayleigh line confirms that the broader central Raman peak observed in Figure 3d of the main text for both chalcohalide films is not an instrumental artefact arising from Rayleigh scattering or filter leakage.

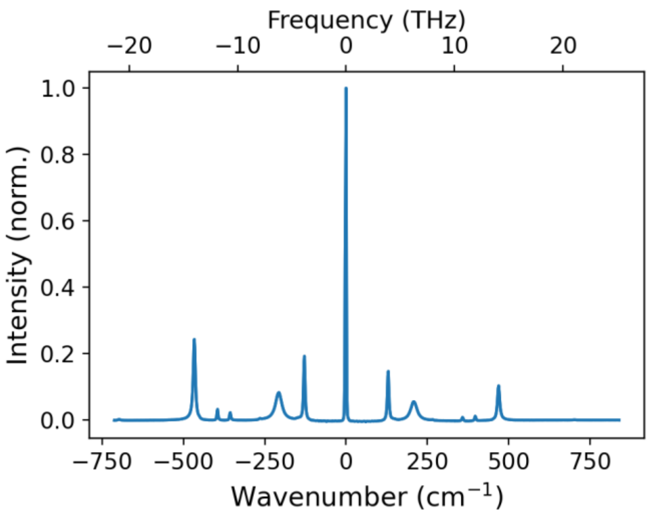

Figure S11 | Normalised Raman spectrum of a z-cut quartz substrate used in this study. Rayleigh scatter was suppressed with volume-Bragg notch filters.

9. References

(1) Manna, D.; Mokurala, K.; Grandhi, G. K.; Al-Anesi, B.; Viswanath, N. S. M.; Sugathan, V.; Tewari, A.; Doyranli, C.; Vivo, P. Solution-Processed Tin-Antimony Quaternary Chalcohalides for Self-Powered Broadband Photodetectors. *Solar RRL* **2024**, *8* (23), 2400633. https://doi.org/10.1002/solr.202400633.

(2) Nie, R.; Lee, K. S.; Hu, M.; Paik, M. J.; Seok, S. Il. Heteroleptic Tin-Antimony Sulfoiodide for Stable and Lead-Free Solar Cells. *Matter* **2020**, *3* (5), 1701–1713. https://doi.org/10.1016/j.matt.2020.08.020.

(3) Righetto, M.; Caicedo-Dávila, S.; Sirtl, M. T.; Lim, V. J.-Y.; Patel, J. B.; Egger, D. A.; Bein, T.; Herz, L. M. Alloying Effects on Charge-Carrier Transport in Silver–Bismuth Double Perovskites. *J. Phys. Chem. Lett.* **2023**, *14* (46), 10340–10347. https://doi.org/10.1021/acs.jpclett.3c02750.

(4) Seifert, T.; Jaiswal, S.; Martens, U.; Hannegan, J.; Braun, L.; Maldonado, P.; Freimuth, F.; Kronenberg, A.; Henrizi, J.; Radu, I.; Beaurepaire, E.; Mokrousov, Y.; Oppeneer, P. M.; Jourdan, M.; Jakob, G.; Turchinovich, D.; Hayden, L. M.; Wolf, M.; Münzenberg, M.; Kläui, M.; Kampfrath, T. Efficient Metallic Spintronic Emitters of Ultrabroadband Terahertz Radiation. *Nat. Photonics* **2016**, *10* (7), 483–488. https://doi.org/10.1038/nphoton.2016.91.

(5) Motti, S. G.; Kober-Czerny, M.; Righetto, M.; Holzhey, P.; Smith, J.; Kraus, H.; Snaith, H. J.; Johnston, M. B.; Herz, L. M. Exciton Formation Dynamics and Band-Like Free Charge-Carrier Transport in 2D Metal Halide Perovskite Semiconductors. *Adv. Funct. Mater.* **2023**, *33* (32), 2300363. https://doi.org/10.1002/adfm.202300363.

(6) Lim, V. J.-Y.; Righetto, M.; Yan, S.; Patel, J. B.; Siday, T.; Putland, B.; McCall, K. M.; Sirtl, M. T.; Kominko, Y.; Peng, J.; Lin, Q.; Bein, T.; Kovalenko, M.; Snaith, H. J.; Johnston, M. B.; Herz, L. M. Contrasting Ultra-Low Frequency Raman and Infrared Modes in Emerging Metal Halides for Photovoltaics. *ACS Energy Lett.* **2024**, *9* (8), 4127–4135. https://doi.org/10.1021/acsenergylett.4c01473.

(7) Haward, T. B.; Lim, V. J.-Y.; Cherniukh, I.; Bodnarchuk, M. I.; Kovalenko, M. V; Herz, L. M. Correlated Vibrational and Electronic Signatures of Surface Disorder in $CsPbBr_3$ Nanocrystals. *ACS Nano* **2025**, *19* (46), 40159–40169. https://doi.org/10.1021/acsnano.5c16045.

(8) Wehrenfennig, C.; Eperon, G. E.; Johnston, M. B.; Snaith, H. J.; Herz, L. M. High Charge Carrier Mobilities and Lifetimes in Organolead Trihalide Perovskites. *Advanced Materials* **2014**, *26* (10), 1584–1589. https://doi.org/10.1002/adma.201305172.

(9) Xia, C. Q.; Peng, J.; Poncé, S.; Patel, J. B.; Wright, A. D.; Crothers, T. W.; Uller Rothmann, M.; Borchert, J.; Milot, R. L.; Kraus, H.; Lin, Q.; Giustino, F.; Herz, L. M.; Johnston, M. B. Limits to Electrical Mobility in Lead-Halide Perovskite Semiconductors. *J. Phys. Chem. Lett.* **2021**, *12* (14), 3607–3617. https://doi.org/10.1021/acs.jpclett.1c00619.

(10) Wright, A. D.; Buizza, L. R. V; Savill, K. J.; Longo, G.; Snaith, H. J.; Johnston, M. B.; Herz, L. M. Ultrafast Excited-State Localization in $Cs_2AgBiBr_6$ Double Perovskite. *J. Phys. Chem. Lett.* **2021**, *12* (13), 3352–3360. https://doi.org/10.1021/acs.jpclett.1c00653.

(11) Buizza, L. R. V; Wright, A. D.; Longo, G.; Sansom, H. C.; Xia, C. Q.; Rosseinsky, M. J.; Johnston, M. B.; Snaith, H. J.; Herz, L. M. Charge-Carrier Mobility and Localization in

Semiconducting Cu_2AgBiI_6 for Photovoltaic Applications. *ACS Energy Lett.* **2021**, *6* (5), 1729–1739. https://doi.org/10.1021/acsenergylett.1c00458.

(12) Buizza, L. R. V; Sansom, H. C.; Wright, A. D.; Ulatowski, A. M.; Johnston, M. B.; Snaith, H. J.; Herz, L. M. Interplay of Structure, Charge-Carrier Localization and Dynamics in Copper-Silver-Bismuth-Halide Semiconductors. *Adv. Funct. Mater.* **2022**, *32* (6), 2108392. https://doi.org/10.1002/adfm.202108392.

(13) Righetto, M.; Wang, Y.; Elmestekawy, K. A.; Xia, C. Q.; Johnston, M. B.; Konstantatos, G.; Herz, L. M. Cation-Disorder Engineering Promotes Efficient Charge-Carrier Transport in $AgBiS_2$ Nanocrystal Films. *Advanced Materials* **2023**, *35* (48), 2305009. https://doi.org/10.1002/adma.202305009.

(14) Lal, S.; Righetto, M.; Ulatowski, A. M.; Motti, S. G.; Sun, Z.; MacManus-Driscoll, J. L.; Hoye, R. L. Z.; Herz, L. M. Bandlike Transport and Charge-Carrier Dynamics in BiOI Films. *J. Phys. Chem. Lett.* **2023**, *14* (29), 6620–6629. https://doi.org/10.1021/acs.jpclett.3c01520.

(15) Lal, S.; Righetto, M.; Putland, B. W. J.; Sansom, H. C.; Motti, S. G.; Jin, H.; Johnston, M. B.; Snaith, H. J.; Herz, L. M. The Role of Chemical Composition in Determining the Charge-Carrier Dynamics in $(AgI)_x(BiI_3)_y$ Rudorffites. *Adv. Funct. Mater.* **2024**, *34* (32), 2315942. https://doi.org/10.1002/adfm.202315942.

(16) Irie, H.; Watanabe, Y.; Hashimoto, K. Nitrogen-Concentration Dependence on Photocatalytic Activity of TiO_2-XN_x Powders. *J. Phys. Chem. B* **2003**, *107* (23), 5483–5486. https://doi.org/10.1021/jp030133h.

(17) Goswami, P.; Ganguli, J. N. Tuning the Band Gap of Mesoporous Zr-Doped TiO_2 for Effective Degradation of Pesticide Quinalphos. *Dalton Transactions* **2013**, *42* (40), 14480–14490. https://doi.org/10.1039/C3DT51891D.

(18) Asahi, R.; Morikawa, T.; Ohwaki, T.; Aoki, K.; Taga, Y. Visible-Light Photocatalysis in Nitrogen-Doped Titanium Oxides. *Science (1979).* **2001**, *293* (5528), 269–271. https://doi.org/10.1126/science.1061051.

(19) Butler-Caddle, E.; Jayawardena, K. D. G. I.; Wijesekara, A.; Milot, R. L.; Lloyd-Hughes, J. Distinguishing Carrier Transport and Interfacial Recombination at Perovskite/Transport-Layer Interfaces Using Ultrafast Spectroscopy and Numerical Simulation. *Phys. Rev. Appl.* **2024**, *22* (2), 24013. https://doi.org/10.1103/PhysRevApplied.22.024013.

(20) Herz, L. M.; Silva, C.; Grimsdale, A. C.; Müllen, K.; Phillips, R. T. Time-Dependent Energy Transfer Rates in a Conjugated Polymer Guest-Host System. *Phys. Rev. B* **2004**, *70* (16), 165207. https://doi.org/10.1103/PhysRevB.70.165207.
